# Antidiabetic Medication Utilisation before and during Pregnancy in Switzerland between 2012 and 2019: An Administrative Claim Database from the MAMA Cohort

**DOI:** 10.1155/2023/4105993

**Published:** 2023-05-10

**Authors:** Eva Gerbier, Guillaume Favre, Emeline Maisonneuve, Michael Ceulemans, Ursula Winterfeld, Kim Dao, Christian P. R. Schmid, Stephen P. Jenkinson, Bartlomiej Niznik, David Baud, Julia Spoendlin, Alice Panchaud

**Affiliations:** ^1^Service of Pharmacy, Lausanne University Hospital and University of Lausanne, 1011 Lausanne, Switzerland; ^2^Materno-Fetal and Obstetrics Research Unit, Department “Woman-Mother-Child”, Lausanne University Hospital, 1011 Lausanne, Switzerland; ^3^Institute of Primary Health Care (BIHAM), University of Bern, 3012 Bern, Switzerland; ^4^Teratology Information Service, Pharmacovigilance Centre Lareb, 's-Hertogenbosch, 5237 MH Hertogenbosch, Netherlands; ^5^Department of Pharmaceutical and Pharmacological Sciences, KU Leuven, 3000 Leuven, Belgium; ^6^L-C&Y, KU Leuven Child and Youth Institute, 3000 Leuven, Belgium; ^7^Swiss Teratogen Information Service and Clinical Pharmacology Service, Lausanne University Hospital, 1011 Lausanne, Switzerland; ^8^Christian P.R. Schmid, CSS Institute for Empirical Health Economics, 6002 Lucerne, Switzerland; ^9^Department of Economics, University of Bern, 3012 Bern, Switzerland; ^10^Hospital Pharmacy, University Hospital Basel, Basel, Switzerland; ^11^Basel Pharmacoepidemiology Unit, Division of Clinical Pharmacy and Epidemiology, Department of Pharmaceutical Sciences, University of Basel, Basel, Switzerland

## Abstract

**Background:**

The incidence of diabetes mellitus (both pregestational and gestational) is increasing worldwide, and hyperglycemia during pregnancy is associated with adverse pregnancy outcomes. Evidence on the safety and efficacy of metformin during pregnancy has accumulated resulting in an increase in its prescription in many reports.

**Aims:**

We aimed to determine the prevalence of antidiabetic drug use (insulins and blood glucose-lowering drugs) before and during pregnancy in Switzerland and the changes therein during pregnancy and over time.

**Methods:**

We conducted a descriptive study using Swiss health insurance claims (2012-2019). We established the MAMA cohort by identifying deliveries and estimating the last menstrual period. We identified claims for any antidiabetic medication (ADM), insulins, blood glucose-lowering drugs, and individual substances within each class. We defined three groups of pattern use based on timing of dispensation: (1) dispensation of at least one ADM in the prepregnancy period and in or after trimester 2 (T2) (pregestational diabetes); (2) dispensation for the first time in or after T2 (GDM); and (3) dispensation in the prepregnancy period and no dispensation in or after T2 (discontinuers). Within the pregestational diabetes group, we further defined continuers (dispensation for the same group of ADM) and switchers (different ADM group dispensed in the prepregnancy period and in or after T2).

**Results:**

MAMA included 104,098 deliveries with a mean maternal age at delivery of 31.7. Antidiabetic dispensations among pregnancies with pregestational and gestational diabetes increased over time. Insulin was the most dispensed medication for both diseases. Between 2017 and 2019, less than 10% of pregnancies treated for pregestational diabetes continued metformin rather than switching to insulin. Metformin was offered to less than 2% of pregnancies to treat gestational diabetes (2017-2019).

**Conclusion:**

Despite its position in the guidelines and the attractive alternative that metformin represents to patients who may encounter barriers with insulin therapy, there was reluctance to prescribe it.

## 1. Introduction

Diabetes mellitus during pregnancy may either refer to pregestational diabetes mellitus or gestational diabetes mellitus (GDM). GDM refers to any degree of glucose intolerance that is first diagnosed during pregnancy, whereas pregestational diabetes is defined as diabetes mellitus (DM) (type 1 or 2) present before conception [1]. Avoidance of maternal hyperglycemia is essential in pregnancies complicated by diabetes mellitus, as it increases the risk of adverse pregnancy outcomes, including major fetal abnormalities, preeclampsia, fetal macrosomia, and neonatal hypoglycemia, amongst others [1, 2]. A linear relationship between maternal blood glucose level during pregnancy and the risk of developing these fetal and maternal complications was shown in the Hyperglycemia and Adverse Pregnancy Outcome (HAPO) study that included 23,316 pregnant women recruited between 2000 and 2006 [3].

The incidence of diabetes mellitus during pregnancy (both pregestational and GDM) has increased worldwide over the last decades and is expected to keep increasing, considering the rise in maternal age at birth and the prevalence of obesity among women of childbearing age [4–6]. Moreover, the implementation of the International Association of the Diabetes and Pregnancy Study Groups (IADPSG) screening criteria has turned an individual risk-based screening strategy of GDM (selective screening) into a universal one, thus increasing the number of diagnosed cases [7, 8]. As a possible result, the use of antidiabetic drugs during pregnancy increased in a retrospective observational study of pregnancies recorded in national databases of several Northern Europe countries, Australia, and the US, between 2006 and 2016 (e.g., from 1.9% to 3.4% in Finland; from 1.3% to 2.0% in Norway; and from 3.0% to 3.9% in the US) [9].

Historically, insulin was the first-line treatment of both pregestational and GDM during pregnancy since it is one of the few drugs that do not cross the placenta and is effective for the attainment of adequate glucose control [10]. Over the last two decades, evidence on the safety and efficacy of metformin use during pregnancy has accumulated, causing a shift in the recommendations of pharmacological treatment of both pregestational and GDM. Results from the MiG trial, a randomized controlled trial of more than 700 pregnant women with GDM in New Zealand and Australia who either received metformin or insulin, showed that metformin was not associated with an increased risk of neonatal complications (neonatal hypoglycemia, acute respiratory distress syndrome, need for phototherapy, and birth trauma) or maternal complications (excessive gestational weight gain, poor glycemic control, and hypertension-related complications) compared to insulin [11]. In 2008, the UK National Institute for Health and Care Excellence (NICE) included metformin in its guidelines as an adjunct or alternative therapy to insulin to treat both pregestational and GDM [12]. Since then, the safety and efficacy profile of metformin (including metformin use after the first trimester) has been reinforced [13–16]. In 2015, the NICE reinforced metformin's position in GDM treatment, placing it as a first-line therapy when diet and exercise are insufficient to achieve glycemic control within 1 to 2 weeks in women with a fasting plasma blood glucose level<7 mmol/L [17]. Other organisations, such as the American College of Obstetricians and Gynecologists (ACOG), do not endorse metformin use as a first-line therapy in GDM [18], possibly due to the metabolic consequences reported in children exposed to metformin in utero (lower birth weight and postnatal accelerated growth and higher mid-childhood body mass index) [19, 20], although this evidence is conflicting [21]. Swiss recommendations align with those provided by the ACOG [22]. Nonetheless, many studies have reported an increase in the use of metformin during pregnancy over the past few years [9, 23]. However, no study has comprehensively explored the use of antidiabetic medications before and during pregnancy in Switzerland. This study is aimed at determining the prevalence of antidiabetic drug use before and during pregnancy in Switzerland and the changes therein during pregnancy and over time.

## 2. Materials and Methods

### 2.1. Data Source

CSS health insurance (Chrétienne sociale suisse Assurance) covers approximately 17% of the Swiss population [24]. The CSS claims data warehouse provides anonymized information on inpatient and outpatient services as well as claims for prescribed medications in the outpatient setting. Indeed, medications dispensed inside the hospital are billed within a fixed rate determined by the SwissDRG tarification system. This system typically takes into account the patient's primary and secondary diagnosis, treatment, and severity. Thus, inpatient prescriptions are not accessible for analysis. Data were extracted for all women with one or more deliveries between January 2012 and December 2019 and continuously covered by CSS during the study observation period which was 9 months before the estimated last menstrual period (LMP) and 9 months after the delivery code for each retraced pregnancy.

### 2.2. Establishment of the MAMA Cohort

#### 2.2.1. Identification of Pregnancies

We identified all inpatient and outpatient pregnancies recorded through SwissDRG (Diagnosis Related Groups system) and TARMED (Swiss pricing system for medical services) delivery codes. Supplementary Table 1 provides all relevant codes used to identify a delivery (stillbirth or livebirth). Pregnancies ending with a miscarriage or an elective/medical termination code were excluded since the aim of the study was to look at longitudinal patterns of medication use.

#### 2.2.2. Identification of Delivery Dates

Multiple delivery codes recorded within 30 days of each other were considered as pertaining to the same pregnancy [25]. The delivery date was set as the first recorded code within this 30-day period, except when both DRG and TARMED codes were recorded, then the DRG code was prioritized and determined as the delivery date. DRG delivery codes which were recorded between 30 and 300 days after an initial date of delivery were ignored (*n* = 108 deliveries), unless the initial delivery was determined by a TARMED code followed by a DRG code, in which case the delivery date was moved to the DRG code. Separate delivery dates had to be recorded at least 300 days apart.

#### 2.2.3. Identification of the Beginning of Pregnancy and Pregnancy Periods

Since neither gestational age nor the beginning of pregnancy are recorded within CSS data, the beginning of pregnancy was estimated using an algorithm, which has previously been validated in US claims data [26]. This method has previously been used also in a Swiss pregnancy cohort based on data from another Swiss health insurance, Helsana [25, 27, 28]. The beginning of pregnancy was set at 270 days before the delivery date for pregnancies ending in a full-term delivery (determined based on DRG codes). Otherwise, the beginning of pregnancy was set at 245 days for pregnancies which had a DRG code indicating a preterm delivery (see Supplementary Table 2). Each pregnancy trimester (Trimester 1 (T1), Trimester 2 (T2), and Trimester 3 (T3)) was defined as a 90-day period, whereby T3 was shortened in case of preterm delivery. We further defined a prepregnancy baseline period covering the 252 days (9 months) preceding the LMP.

### 2.3. Antidiabetic Medication Exposure

We captured exposure to any antidiabetic medication (ADM) based on recorded Anatomical Therapeutic Classification (ATC) codes for *Drugs used in diabetes* (A10). We also captured exposure to the following individual antidiabetic drug classes: *insulins and analogues* (A10A), *blood glucose-lowering drugs* (A10B), as well as individual drug substances within each drug group. Blood glucose-lowering drugs included both oral hypoglycemic agents and non-insulin injectable glucose-lowering medications. Exposure was defined as at least one dispensation of an ADM in an outpatient setting during the periods of interest (i.e., prepregnancy period, T1, T2, and T3).

### 2.4. Study Groups

We defined three main groups of ADM users during pregnancy: (1) with dispensation in the prepregnancy period and in or after T2 referred hereafter to as ADM for pregestational diabetes mellitus; (2) with a dispensation for the first time in or after T2 referred hereafter to as ADM for GDM; and (3) with a dispensation in the prepregnancy period and no dispensation in or after T2 referred to as discontinuers. With regard to ADM use in the pregestational diabetes mellitus group, we subdivided pregnancies in two treatment patterns: continuers and switchers. Continuers were defined as pregnancies with a dispensation for the same group of ADM in the prepregnancy period and in or after T2. Switchers were defined as pregnancies with a dispensation of a specific group of ADM in the prepregnancy period and with a switch to a different ADM group in or after T2.

### 2.5. Covariates

For each pregnancy, demographic information was extracted, including the year of delivery and the mother's age at delivery.

### 2.6. Statistical Analysis

We quantified the prevalence of exposure to at least one ADM, insulin product, blood glucose-lowering drug, and individual antidiabetic substances, within the above-described groups (i.e., pregestational diabetes mellitus continuers and pregestational diabetes mellitus switchers, GDM, and discontinuers). Exposure prevalence was calculated over the entire study period as well as by calendar year. Prevalence of exposure was defined as the proportion of pregnancies during which at least one prescription was filled for the respective active substance, divided by the total number of enrolled pregnancies during the respective period. Results are presented as absolute numbers divided by 10000 with 95% confidence intervals.

All analyses were performed using the statistical software R studio (version 1.2.5001).

## 3. Results

We identified a population of 104,098 deliveries from 80,320 women. The mean maternal age at delivery in our cohort was 31.7 years. In total, 31.9% of all deliveries were caesarean sections (see Table 1).

### 3.1. Pregnancies in which an ADM Was Dispensed Either during the Prepregnancy Period and/or Pregnancy between 2012 and 2019 in the MAMA Cohort

**Figure 1:**
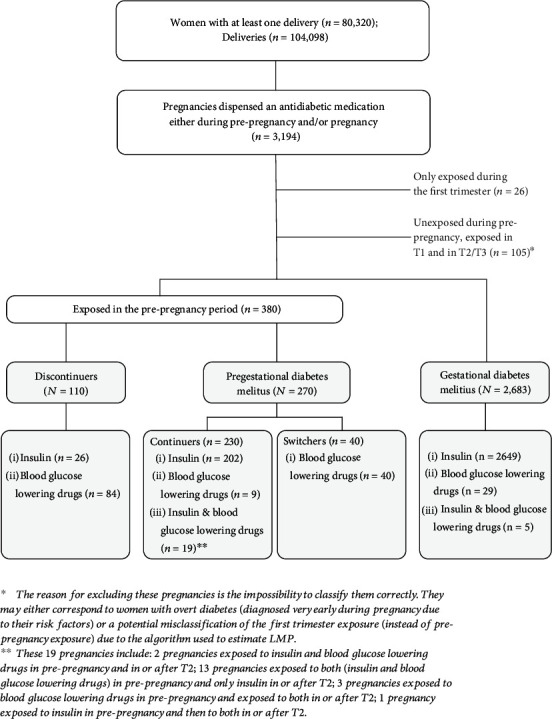
Different ADM dispensing patterns among pregnancies prescribed at least one ADM during the prepregnancy period and/or during pregnancy (i.e., pregestational diabetes mellitus, GDM, and discontinuers). Data refer to calendar years between 2012 and 2019.

Figure 1 describes the different ADM dispensing patterns among pregnancies in which at least one ADM was prescribed during the prepregnancy period and/or during pregnancy (i.e., pregestational diabetes mellitus, GDM, and discontinuers). Data refer to calendar years between 2012 and 2019.

### 3.2. Patterns of Medication Use among Pregnancies Exposed to an ADM during the Prepregnancy Period between 2012 and 2019

In total, at least one ADM was dispensed to 36.5/10000 pregnancies during the 9 months preceding pregnancy between 2012 and 2019, including at least one insulin product to 22.0/10000 (22.0/36.5, 60.3%) pregnancies, at least one blood glucose-lowering drug to 13.1/10000 (13.1/36.5, 35.9%) pregnancies, and both an insulin product and a blood glucose-lowering drug to 1.4/10000 (1.4/36.5, 3.8%) pregnancies. Among these pregnancies, 25.9/10000 (25.9/36.5, 71.0%) were also dispensed an ADM in or after T2 defined as ADM for pregestational diabetes mellitus. The prevalence of ADM prescriptions for pregestational diabetes mellitus increased between 2012 (24.4/10000, 95% CI 16.0-35.8) and 2019 (30.7/10000, 95% CI 22.4-41.1). Among pregnancies in which an ADM was dispensed for pregestational diabetes mellitus between 2012 and 2019, 22.1/10000 (22.1/25.9, 85.2%) continued the use of the same ADM (continuers) and 3.8/10000 (3.8/25.9, 14.8%) switched to a different ADM (switchers).

In total, 10.7/10000 (10.7/36.5, 29.1%) pregnancies in which an ADM was dispensed before pregnancy discontinued their treatment (unexposed in T2 and T3), with an increasing prevalence over time between 2012 (4.7/10000, 4.7/29.1; 16.2%, 95% CI 5.6-34.7) and 2019 (21.2/10000, 21.2/51.9; 40.8%, 95% CI 27.0-54.9).

**Figure 2:**
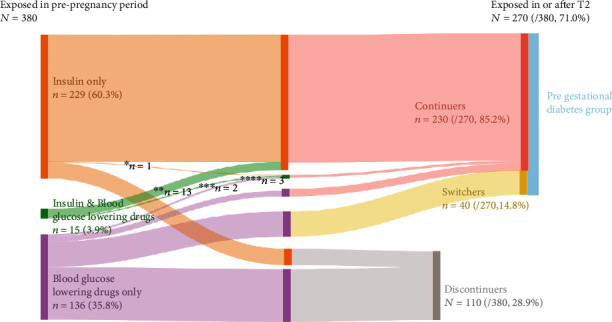
Proportion of pregnancies in which a given ADM was continued, switched, or discontinued, among pregnancies exposed to an ADM during the prepregnancy period between 2012 and 2019. ^∗^Insulin in prepregnancy and then insulin and blood glucose-lowering drugs in or after T2 (*n* = 1); ^∗∗^insulin and blood glucose-lowering drugs in prepregnancy and insulin only in or after T2 (*n* = 13); ^∗∗∗^insulin and blood glucose-lowering drugs both in prepregnancy and in or after T2 (*n* = 2); ^∗∗∗∗^blood glucose-lowering drugs in prepregnancy and blood glucose-lowering drugs + insulin in or after T2 (*n* = 3).

Figure 2 presents the proportion of pregnancies in which a given ADM was continued, switched, or discontinued, among pregnancies exposed to an ADM during the prepregnancy period between 2012 and 2019. Supplementary Table 3 describes the prevalence of exposure to insulin, to blood glucose-lowering drug, or to both insulin and blood glucose-lowering drugs, among continuers, switchers, and discontinuers during the prepregnancy period. Data refer to the overall study period (2012–2019) and per year between 2012 and 2019.

**Figure 3:**
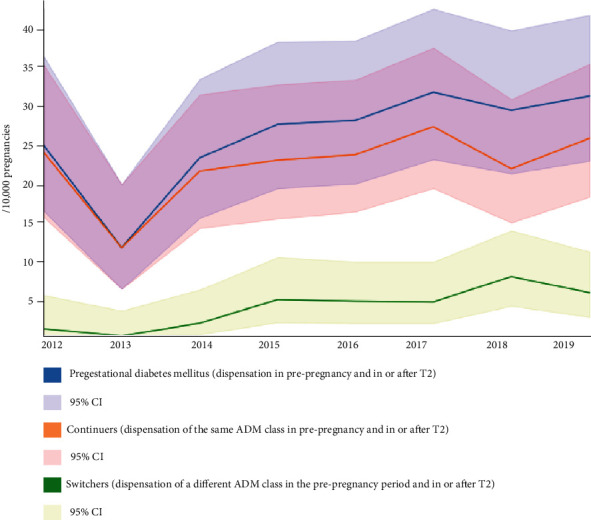
Prevalence of pregnancies in which an ADM was dispensed in the group of pregestational diabetes mellitus (with continuers and switchers within this group). Data refer to calendar years between 2012 and 2019.

Figure 3 shows the prevalence of pregnancies in which an ADM was dispensed in the group of pregestational diabetes mellitus (with continuers and switchers within this group). Data refer to calendar years between 2012 and 2019.

### 3.3. Prevalence of Different Types of ADM Regimen among Pregnancies in which an ADM Was Dispensed for Pregestational Diabetes Mellitus

#### 3.3.1. Continuers

Among pregnancies in which at least one ADM was dispensed during the prepregnancy period and in or after T2 between 2012 and 2019 (25.9/10000), 22.1/10000 (22.1/25.9, 85.3%) continued the use of the same ADM. This included 19.5/10000 (19.5/22.1, 88.2%) pregnancies dispensed insulin only, 1.4/10000 (1.4/22.1, 6.5%) dispensed both insulin and a blood glucose-lowering drug in the prepregnancy period (and to both or either one during or after T2), and 1.2/10000 (1.2/22.1, 5.2%) a blood glucose-lowering drug only. The proportion of insulin continuers decreased between 2012 (21.6/10000, 21.6/23.5; 92.0%, 95% CI 73.0-98.9) and 2019 (19.8/10000, 19.8/25.3; 78.3%, 95% CI 59.3-93.2 in 2019) while that of blood glucose-lowering drugs continuers increased (0/10000, 0/23.5; 0.0%, 95% CI 0.0-14.2 in 2012; 3.4/10000, 3.4/25.3; 13.5%, 95% CI 2.5-31.2 in 2019). This tendency seemed especially relevant when comparing blood glucose-lowering drugs continuers between the late years of observation (2017-2019: 7.6/10000, 7.6/73.5; 10.3%, 95% CI 4.8-20.2) to the early years (2012–2016: 0.8/10000, 0.8/101.6, 0.8%, 95% CI 0.0-5.4). The most dispensed insulin in continuers during the prepregnancy period between 2012 and 2019 was insulin aspart (42.3%), followed by insulin lispro (20.9%). The most dispensed blood glucose-lowering drug was metformin (66.1%), followed by glucagon-like peptide 1 (GLP-1) receptor agonists (21.0%) and sodium-glucose cotransporter-2 (SGLT2) inhibitors (8.1%). Supplementary Table 4 and Supplementary Figures 1 and 2 show the distribution of the different insulin and blood glucose-lowering drug prescriptions within each group of pregestational diabetes mellitus (continuers, switchers) during the prepregnancy, overall and per year between 2012 and 2019.

#### 3.3.2. Switchers

Among pregnancies in which at least one ADM was dispensed during the prepregnancy (25.9/10000) and switched to a different ADM in or after T2 (3.8/10000) between 2012 and 2019, all were dispensed a blood glucose-lowering drug during the prepregnancy and switched to insulin during pregnancy (no pregnancy was switched from insulin to a blood glucose-lowering drug). Among pregnancies exposed to a blood glucose-lowering drug in the prepregnancy period and exposed to an ADM in or after T2 (5.0/10000) between 2012 and 2019, there was a switch to insulin during pregnancy in 3.8/10000 (3.8/5.0, 76.0%) pregnancies. The relative proportion of blood glucose-lowering drugs switchers decreased over time (0.9/10000, 0.9/0.9; 100%, 95% CI 25-100 in 2012; 5.5/10000, 5.5/8.9; 61.8%, 95% CI 29.9-93.7 in 2019). Among pregnancies in which a blood glucose-lowering drug was prescribed during the prepregnancy period and then switched to insulin during pregnancy, metformin was dispensed to 32.3% of them in the prepregnancy period, followed by dipeptidyl peptidase 4 (DPP-4) inhibitors (22.6%) and GLP-1 receptor agonists (18.0%) (see Supplementary Table 4 and Supplementary Figure 2).

Prevalence of different types of ADM regimen among pregnancies in which the use of any ADM was discontinued between prepregnancy and pregnancy.

#### 3.3.3. Discontinuers

Among pregnancies in which at least one ADM was prescribed during the prepregnancy period, and in which no ADM dispensation was recorded in or after T2 (10.7/10000), a blood glucose-lowering drug was dispensed to 8.1/10000 (8.1/10.7, 75.7%) during the prepregnancy period (88.3% metformin, 8.2% GLP-1 receptor agonists (liraglutide)) and insulin was dispensed to 2.5/10000 (2.5/10.7, 23.3% (36.7% human insulin and 32.7% aspart insulin)). The relative proportion of discontinuers was stable over time both among blood glucose-lowering drugs-exposed pregnancies (1.9/10000, 1.9/2.8; 67.9%, 95% CI 9.4-99.2 in 2012 and 17.8/10000, 17.8/26.6, 66.9%; 95% CI 46.0-83.5 in 2019) and insulin-exposed pregnancies (2.8/10000, 2.8/24.4; 11.5%, 95% CI 2.4-30.0 in 2012 and 3.4/10000, 3.4/23.2; 14.7%, 95% CI 4.8-30.0 in 2019, (see Supplementary Table 3). Among pregnancies in which a blood glucose-lowering drugs was dispensed, 32.1% (27/84) were exposed to a medication used in fertility treatment (see Supplementary Table 5 for ATC codes of medications included as fertility treatment).

Supplementary Table 6 and Supplementary Figure 3 show the distribution of the different insulin and blood glucose-lowering drug prescriptions among discontinuers during the prepregnancy period, overall, and per year between 2012 and 2019.

### 3.4. Prevalence of Different Types of ADM Regimen among Pregnancies in the GDM Group

**Figure 4:**
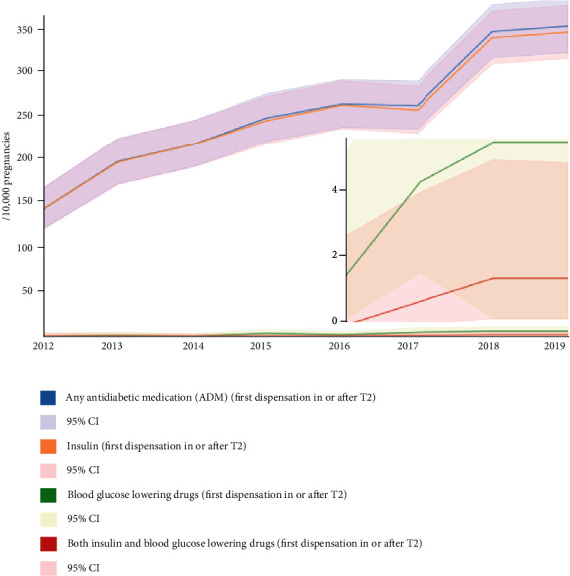
Prevalence of pregnancies exposed to any ADM, an insulin product, a blood glucose-lowering drug, or both an insulin product and a blood glucose-lowering drug, in the GDM group, per calendar year between 2012 and 2019.

Overall, at least one ADM was dispensed for the first time in or after T2 in 257.7/10000 pregnancies, with an increasing prevalence between 2012 (141.9/10000, 1.4%, 95% CI 1.2-1.7) and 2019 (348.3/10000, 0.3%, 95% CI 0.3-0.4). Among these pregnancies, 254.5/10000 (254.5/257.7, 98.8%) were prescribed at least one insulin product, 2.8/10000 (2.8/257.7, 1.1%) at least one blood glucose-lowering drug, and 0.5/10000 (0.5/257.8, 0.2%) both a blood glucose-lowering drug and an insulin product. The proportion of insulin-exposed pregnancies in the GDM group has slightly decreased when comparing the early (2012-2016: 1054.4/10000, 1054.4/1059.7, 99.5%, 95% CI 98.7-99.8) and late years (2017-2019: 931/10000, 931/949.9, 98.0%, 95% CI 95.8-98.1) of observation, while that of blood glucose-lowering drugs-exposed pregnancies in the GDM group has increased (2012-2016: 5.5/10000, 5.5/1059.7, 0.5%, 95% CI 0.2-1.2; 2017-2019: 15.3/949.9, 1.6%, 95% CI 0.8-2.5).  Figure 4 shows the prevalence of pregnancies exposed to any ADM in the GDM group, as well as the prevalence of pregnancies exposed to an insulin product exclusively, a blood glucose-lowering drug exclusively, or both an insulin product and a blood glucose-lowering drug in this group, per year between 2012 and 2019 (see Supplementary Table 7). Supplementary Table 8 and Supplementary Figure 4 show the distribution of the different insulin and blood glucose-lowering drugs prescriptions in the GDM group during or after T2, overall and per year between 2012 and 2019.

## 4. Discussion

This study is aimed at determining the prevalence and patterns of exposure to ADMs before and during pregnancy between 2012 and 2019 in Switzerland using the MAMA cohort. We identified 104,087 deliveries with a mean maternal age at delivery of 31.7 years and 31.9% of caesarean sections. These numbers were in line with those reported by the Swiss Federal Statistical Office for the overall population of women giving birth in Switzerland [29, 30]. Therefore, our study population appears to be representative of the overall Swiss population during this period.

Overall, an ADM was dispensed to 25.9/10000 (0.3%) pregnancies during both the prepregnancy period and in or after T2 between 2012 and 2019, likely reflecting the prevalence of pregestational diabetes mellitus in our cohort. This prevalence increased over the study period (24.4/10000 in 2012; 30.7/10000 in 2019). A previous observational European study based on healthcare databases reported a similar prevalence of pregestational diabetes mellitus in Italy, the Netherlands, Denmark, and the UK between 2004 and 2009, ranging from 0.3% to 0.5% [23]. Within our group of pregestational diabetes mellitus treated with a blood glucose-lowering drug, in most pregnancies there was a switch to insulin before the start of pregnancy during the early observation years (2012-2016), whereas this proportion slightly decreased during later years (2017-2019). On the other hand, the proportion of pregnancies continuing the use of a blood glucose-lowering drug between the prepregnancy period and pregnancy in the pregestational group increased between the early and late observation period, representing approximately 10.3% of all pregnancies treated for pregestational diabetes mellitus between 2017 and 2019. This represented 0.03% of all pregnancies in the cohort, which was similar to the prevalence of metformin continuers reported in a recent Swiss study based on Helsana claims data collected between 2014 and 2018 (0.05%) [25]. Nevertheless, it appears that still a large proportion of women with pregestational diabetes mellitus are being switched to insulin during pregnancy, despite metformin being accepted as an adjunct or alternative therapy for women in Swiss recommendations for clinical practice [31]. Although many of these women likely required an insulin switch to maintain optimal diabetes mellitus control, others might have been able to continue metformin and avoid certain constraints, including the need to learn/adapt to a new treatment, the risk of hypoglycemia and weight gain and the inconvenience/unpleasantness of injections [11].

In total, an ADM was dispensed to 10.7/10000 (0.1%) pregnancies during the prepregnancy period and discontinued the use of any ADM during pregnancy. This represented one-third of all pregnancies exposed to an ADM in the prepregnancy period, similarly to the proportion reported in the European multinational study (11-35%) [23]. Of these pregnancies, 3/4 were dispensed a blood glucose-lowering drug which included 88% of metformin prescriptions. Metformin is often used in the treatment of polycystic ovary syndrome (PCOS) and PCOS-related infertility [32] as well as in obesity [33]. In addition, 1/3 were dispensed other medications which are commonly used as part of infertility treatment (see Supplemental Table 5) [34]. This proportion may be slightly underestimated since it was not possible to identify prescriptions for clomiphene in the MAMA cohort, a treatment among certain women with PCOS-related infertility [34]. Indeed, prescriptions are recorded through Swissmedic numbers and clomiphene was no longer available in Switzerland by the time of analysis (October 2022) and therefore its Swissmedic number no longer existed. One quarter of discontinuers, representing 2.5/10000 (0.03%) of all pregnancies, were dispensed an insulin product before pregnancy but were not dispensed any other ADM during pregnancy. There is no other official indication for insulin than the treatment of diabetes mellitus. However, it is possible that these pregnancies had an off-label use of insulin [35].

Finally, an ADM was dispensed for the first time during or after trimester in 257.7/10000 (2.6%) pregnancies. These women likely reflect pharmacologically treated GDM. This prevalence more than doubled over the study period. Prevalence of pregnancies receiving their first ADM prescription in the 2nd or 3rd trimester in the multinational European study varied between 0.2% in Denmark to 1.9% in Tuscany (Italy) [23]. These lower results were observed before the implementation of the IADPSG criteria [36], which have been in part responsible for the increase in GDM prevalence. For instance, a retrospective Belgian study comparing the prevalence of GDM in women classified according to Carpenter-Coustan or IADPSG criteria between 2009 and 2016 found a dramatic increase in GDM prevalence among IADPSG diagnosed women (net increase in GDM from 8 to 18%) [37]. In Switzerland, expert guidelines adapted from the IADPSG criteria were edited by the Swiss Society of Gynaecology and Obstetrics (SGGO) in 2011, recommending universal screening between 24 and 28 gestational weeks and early screening for women with specific risk criteria [38].

Almost all pregnancies pharmacologically treated for GDM were prescribed insulin exclusively, mostly insulin detemir and human insulin. This is in line with the Swiss recommendations for clinical practice (RCP) stating that both Neutral Protamine Hagedorn (NPH) insulins (i.e., isophane insulin, “insulatard,” or “huminsulin”) and insulin detemir may be used during pregnancy [22]. During the latest years of observation (2017-2019), the proportion of pregnancies in the GDM group which were started on an OHA treatment has increased reaching 1.6% (97% metformin, 3% sulfonylurea). Since 2017, similarly to the ACOG's recommendations [18], Swiss clinical recommendations state that metformin may be used as a second intention treatment for GDM, typically when adherence issues are observed with insulin use [22]. Recently, a randomized clinical trial in Spain assessed glycemic control, obstetrical and perinatal outcomes among 200 pregnant women with GDM either treated with metformin or insulin [39]. Authors observed a similar glycemic control with fewer hypoglycemic events with metformin, less maternal weight gain and a low rate of failure as an isolated treatment compared to insulin or to both insulin and metformin (i.e., patient acceptability and preference of use in a subsequent pregnancy) [39]. Other obstetrical and perinatal outcomes were similar [39]. Furthermore, recent results from the follow-up at 24 months of 283 children exposed either to placebo or metformin in utero during the MiTY trial showed similar anthropometric measurements (body mass index (BMI) and skinfold thickness) [40].

### 4.1. Methodological Considerations

Our group of pregestational diabetes was defined as pregnancies exposed to an ADM both during prepregnancy and in or after T2. The reason for this definition was to exclude women who stopped taking their diabetes medication during pregnancy and were therefore likely taking antidiabetic medication for reasons other than pregestational diabetes, such as polycystic ovary syndrome (PCOS) or infertility (group of discontinuers). Regarding gestational diabetes mellitus (GDM), ideally, we would have liked to distinguish between GDM and overt diabetes during pregnancy. Overt diabetes, defined as a woman who has a glucose level that meets the threshold of diabetes in non-pregnant adults, may be due to preexisting diabetes that was previously unknown or prediabetes that progressed during pregnancy. In Switzerland, some women with overt diabetes may be diagnosed through early screening based on specific criteria (age > 35, previous diagnosis of GDM or macrosomia, BMI > 30, family history of type 2 diabetes, or PCOS). We could have created a group of women with overt diabetes for those with a first prescription after the first trimester. However, some women with overt diabetes may not present any of the above-mentioned risk factors for early screening or may simply not want to participate in it. Thus, we estimated that distinguishing precisely between the two was not possible with the data we had and decided to create only one group. We therefore used the cutoff of a first prescription in or after the second trimester to capture both women with overt diabetes (who might already have a prescription during the second trimester) and women with GDM (who should have a prescription at the end of the second trimester or beginning of the third). One might suggest that we only capture women in the third trimester, as those who received a prescription in the second trimester are likely to continue receiving one in the third. However, the number of times a woman needs to refill her prescription can vary based on factors like medication type, dosage, and packaging. Therefore, if we had set our cutoff after the 24th week, it is possible that some women who had started their medication before this time may have already obtained enough medication to last beyond our cutoff and would not have been included in our analysis.

### 4.2. Strengths and Limitations

To our knowledge, this study is the first to evaluate utilisation of ADM before and during pregnancy over time on a population-based level in Switzerland. Our results are based on large administrative database covering 17% of the Swiss population in 2021, the MAMA cohort. However, the lack of information on the sociodemographic and anthropometric characteristics (BMI) of included women prevents us from generalizing our results to the entire population of Swiss pregnant women. Nevertheless, maternal age at delivery has been shown to be a reliable proxy for socioeconomic status [41], which is in turn associated with overweight and obesity [42]. Since maternal age in our study population aligns with numbers reported by the Bundesamt für Statistik **(**BfS), we believe our results were not substantially affected by channeling.

Since information on medication dispensations was recorded automatically as a byproduct of routine pharmacy care, our results are not susceptible to maternal volunteer or recall bias. Still, it is not possible to know whether the medications were actually taken by the women and whether they were taken close to the time of dispensation. In addition, gestational age and trimesters were calculated based on an algorithm which has been validated in US claims data but not in Swiss data, and thus, we cannot exclude the possibility of some exposure misclassification. However, given that we focused on rather long periods of interest (either the entire pregnancy, 9 months prepregnancy, or semesters) instead of specific trimesters, this risk should have been minimized. Our data only included information on ambulatory dispensations, and we did not have information on inpatient medication use. However, we believe that this limitation is not likely to have affected the prevalence of exposure to antidiabetic medications in the study, as these medications are typically prescribed and dispensed in an outpatient setting. Therefore, missing information on inhospital prescriptions is not expected to have a significant impact on the study's findings related to antidiabetic medication use. Finally, we did not include pregnancies resulting in a miscarriage or an elective/medical abortion since the aim of the study was to look at longitudinal patterns of antidiabetic medication use. However, we do not believe that our prevalence of antidiabetic use would have been influenced by the inclusion of these women since neither insulin nor metformin (which represent almost all antidiabetic use in our cohort) have been associated with a higher risk of miscarriage [43].

## 5. Conclusion

In this study, the increased dispensations of antidiabetic medications among women with pregestational diabetes mellitus and GDM in Switzerland reflect the worldwide rise in the prevalence of diabetes mellitus among women of childbearing age. Insulin was the most frequently dispensed medication to treat both diseases. However, between 2017 and 2019, women with a pregestational blood glucose-lowering drug (mostly metformin) increasingly continued the same treatment throughout pregnancy rather than switching to insulin (10% of all pregnancies treated for pregestational diabetes mellitus). Nonetheless, this still accounted for a much smaller proportion than women who switched treatment. Most women (>98%) with GDM were treated with insulin, and very few women seem to have been offered metformin as an alternative in most recent years (2017-2019). The pattern of use of metformin suggests that there is a reluctance to prescribe it, despite its position in the guidelines. However, it offers an attractive alternative for patients for whom the introduction of insulin therapy represents a barrier, and the latest evidence is reassuring regarding the long-term health of children exposed in utero.

## Figures and Tables

**Table 1:** Description of the MAMA cohort population.

Year	MAMA cohort			Swiss statistics from the Bundesamt für Statistik (BfS) [29, 30]		
	No. of deliveries	Mean maternal age at delivery (years (min-max))	Caesarean section (*n*, %)^1^	No. of deliveries^2^	Mean maternal age at delivery (years)	Caesarean section (*n*, %)
2012^∗^	10,639	31.3 (14.5-46.4)	3,431 (32.2)	81,274	31.5	27,115 (33.4)
2013	11,484	31.4 (14.2-47.3)	3,742 (32.6)	81,951	31.6	27,310 (33.3)
2014	12,306	31.6 (15.5-48.5)	3,940 (32.0)	84,014	31.7	23,337 (33.3)
2015	12,917	31.6 (15.7-48.9)	4,243 (32.8)	85,421	31.8	28,483 (33.3)
2016	13,780	31.8 (15.1-50.3)	4,460 (32.4)	86,787	31.8	28,778 (33.2)
2017	13,803	31.9 (15.7-51.4)	4,303 (31.2)	85,990	31.9	27,814 (32.3)
2018	14,525	32.0 (15.9-50.7)	4,574 (31.5)	86,411	32.0	27,754 (32.1)
2019	14,644	32.2 (16.2-51.8)	4,558 (31.1)	85,128	32.1	27,246 (32.0)
2012-2019	104,098^∗∗^	31.7 (14.2-51.8)	33,247 (31.9)	676,976	31.8	217,837 (32.1)

^1^In total, there were 14 deliveries recorded as neonatal deaths without a code specifying the mode of delivery. ^2^ Includes liveborn infants only. ^∗^Before 01.01.2012, recorded SwissDRG and TARMED codes did not correspond to the codes we used to identify deliveries (example of codes which were recorded before 2012 but were not counted as deliveries: “O64A”, “O64B”, and “O61Z”). ^∗∗^104,098 pregnancies recorded from 80,320 women.

## Data Availability

The data that support the findings of this study were used under license for the current study and so are not publicly available. Data are however available from the authors upon reasonable request and with permission of the data provider.

## Abbreviations

GDM: Gestational diabetes mellitus
DM: Diabetes mellitus
HAPO: Hyperglycemia and Adverse Pregnancy Outcome
IADPSG: International Association of the Diabetes and Pregnancy Study Groups
NICE: National Institute for Health and Care Excellence
ACOG: American College of Obstetricians and Gynecologists
LMP: Last menstrual period
SwissDRG: Diagnosis Related Groups system
TARMED: Swiss pricing system for medical services
ADM: Antidiabetic medication
ATC: Anatomical Therapeutic Classification
T1: Trimester 1
T2: Trimester 2
T3: Trimester 3.

## Supplementary Materials

Supplementary Materials Supplemental Table 1: relevant codes used to identify a delivery. Supplemental Table 2: relevant codes to identify a preterm delivery. Supplemental Table 3: prevalence of exposure to insulin, to blood glucose-lowering drugs, or to both insulin and blood glucose-lowering drugs, among continuers, switchers, and discontinuers during the prepregnancy period, overall and per year between 2012 and 2019. Supplemental Table 4: proportion of different insulin and blood glucose-lowering drugs prescriptions within each group of pregestational diabetes mellitus (continuers, switchers) during the prepregnancy period, overall and per year between 2012 and 2019. Supplemental Table 5: ATC codes of medication used in the treatment of infertility. Supplemental Table 6: proportion of different insulin and blood glucose-lowering drugs prescriptions among discontinuers, overall and per year between 2012 and 2019. Supplemental Table 7: prevalence of pregnancies exposed to any ADM, to insulin, to a blood glucose-lowering drugs, or to both insulin and a blood glucose-lowering drug, in the gestational diabetes mellitus group, overall and per year between 2012 and 2019. Supplemental Table 8: proportion of different insulin and blood glucose-lowering drugs prescriptions in the gestational diabetes mellitus group during or after T2, overall and per year between 2012 and 2019. Supplemental Figure 1: distribution of the different insulin and blood glucose-lowering drugs prescriptions among continuers during the prepregnancy period per calendar year between 2012 and 2019. Supplemental Figure 2: distribution of the different blood glucose-lowering drugs prescriptions among switchers during the prepregnancy period per calendar year between 2012 and 2019. Supplemental Figure 3: distribution of the different insulin and blood glucose-lowering drugs prescriptions among discontinuers during the prepregnancy period per calendar year between 2012 and 2019. Supplemental Figure 4: distribution of the different insulin and blood glucose-lowering drugs prescriptions within the gestational diabetes group during or after T2 per calendar year between 2012 and 2019.
