# Supplementary material for: Antidiabetic Medication Utilisation before and during Pregnancy in Switzerland between 2012 and 2019: An Administrative Claim Database from the MAMA Cohort
Source: J Diabetes Res. 2023 May 10;2023:4105993. doi: 10.1155/2023/4105993 (PMC10191745; doi:10.1155/2023/4105993)

**Supplementary materials**

Supplementary Tables:

*Supplemental Table 1: Relevant codes used to identify a delivery.*

*Supplemental Table 2: Relevant codes to identify a preterm delivery.*

*Supplemental Table 3:* Prevalence of exposure to insulin, to blood glucose lowering drugs, or to both insulin and blood glucose lowering drugs, among continuers, switchers, and discontinuers during the pre-pregnancy period, overall and per year between 2012 and 2019.

*Supplemental Table 4: Proportion of different insulin and* blood glucose lowering drugs *prescriptions within each group of pregestational diabetes mellitus (continuers, switchers) during the pre-pregnancy period, overall and per year between 2012 and 2019.*

*Supplemental Table 5: ATC codes of medication used in the treatment of infertility.*

*Supplemental Table 6: Proportion of different insulin and* blood glucose lowering drugs *prescriptions among discontinuers, overall and per year between 2012 and 2019.*

*Supplemental Table 7: Prevalence of pregnancies exposed to any ADM, to insulin, to a* blood glucose lowering drugs*, or to both insulin and a* blood glucose lowering drug*, in the gestational diabetes mellitus group, overall and per year between 2012 and 2019.*

*Supplemental Table 8: Proportion of different insulin and* blood glucose lowering drugs *prescriptions in the gestational diabetes mellitus group during or after T2, overall and per year between 2012 and 2019.*

Supplementary Figures:

*Supplemental Figure 1: Distribution of the different insulin and* blood glucose lowering drugs *prescriptions among continuers during the pre-pregnancy period per calendar year between 2012 and 2019.*

*Supplemental Figure 2: Distribution of the different* blood glucose lowering drugs *prescriptions among switchers during the pre-pregnancy period per calendar year between 2012 and 2019.*

*Supplemental Figure 3: Distribution of the different insulin and* blood glucose lowering drugs *prescriptions among discontinuers during the pre-pregnancy period per calendar year between 2012 and 2019.*

*Supplemental Figure 4: Distribution of the different insulin and* blood glucose lowering drugs *prescriptions within the gestational diabetes group during or after T2 per calendar year between 2012 and 2019.*

*Supplemental Table 1: Relevant codes used to identify a delivery.*

|  | **Original codes** | **English translation** |
| --- | --- | --- |
| **TarMed codes version**  (V01.07.02, 01.08.00, 01.08.01, 01.09) | **Grossesse et obstétrique** | **Pregnancy and obstetrics** |
| **22.2110** | Surveillance de la naissance et conduite de l'accouchement, risque normal | Birth and delivery management, normal risk |
| **22.2120** | +Césarienne secondaire | +Secondary caesarean section |
| **22.2130** | +Hystérectomie lors d’une césarienne | +Hysterectomy after caesarean section |
| **22.2200** | Surveillance de la naissance et conduite de l'accouchement, haut risque | Birth and delivery management, high risk |
| **22.2210** | [Surveillance de la naissance et conduite de l'accouchement, très haut risque](https://www.tarmed-browser.ch/fr/prestations/22.2210-surveillance-de-la-naissance-et-conduite-de-l-accouchement-tres-haut-risque) | Birth and delivery management, very high risk |
| **22.2410** | Césarienne, planifiée ou primaire | Caesarean section, planned or primary |
| **22.2420** | Césarienne itérative | Iterative caesarean section |
| **SwissDRG Codes** | **MDC 14: Grossesse, naissance et suites de couches** | **MDC 14: Pregnancy, birth and postnatal care** |
| **O01A** (V 1.0, V 2.0, V3.0, V4.0, V 5.0, V6.0) | Césarienne avec plusieurs diagnostics de complication, durée de la grossesse jusqu'à 25 semaines complètes ou avec thérapie intra-utérine | Caesarean section with multiple complication diagnoses, duration of pregnancy up to 25 completed weeks or with intrauterine therapy |
| **O01A** (V7.0, V8.0, V9.0) | Césarienne et dialyse, ou thérapie intra-utérine complexe du fœtus | Caesarean section and dialysis, or complex intrauterine fetal therapy |
| **O01B** (V 1.0, V 2.0, V3.0, V4.0 | Césarienne avec plusieurs diagnostics de complication, durée de la grossesse de 26 à 33 semaines complètes, sans thérapie intra-utérine ou avec diagnostic de complication, jusqu'à 25 semaines complètes, ou thromboembolie pendant la période de gestation avec procédure opératoire | Caesarean section with several diagnoses of complication, duration of pregnancy from 26 to 33 completed weeks, without intrauterine therapy or with diagnosis of complication, up to 25 completed weeks, or thromboembolism during the gestation period with operative procedure |
| **O01B** (V5.0) | Césarienne avec plusieurs diagnostics de complication, durée de la grossesse de 26 à 33 semaines complètes, jusqu'à 25 semaines complètes, ou thromboembolie pendant la période de gestation avec procédure opératoire ou procédure complexe | Caesarean section with several diagnoses of complication, duration of pregnancy from 26 to 33 completed weeks, up to 25 completed weeks, or thromboembolism during the gestation period with operative procedure or complex procedure |
| **O01B** (V6.0, V7.0) | Césarienne avec plusieurs diagnostics de complication, durée de la grossesse de 26 à 33 semaines ou CC extrêmement sévères ou diagnostic complexe ou procédure de complication, jusqu'à 33 semaines de grossesse ou diagnostic complexe et CC extrêmement sévères, ou jusqu'à 25 semaines de grossesse et diagnostic de complication | Caesarean section with multiple complication diagnoses, duration of pregnancy 26-33 weeks or extremely severe CC or complex diagnosis or complication procedure, up to 33 weeks of pregnancy or complex diagnosis and extremely severe CC, or up to 25 weeks of pregnancy and complication diagnosis |
| **O01B** (V8.0, V9.0) | Césarienne avec plusieurs diagnostics de complication, durée de la grossesse jusqu'à 25 semaines ou avec thérapie intra-utérine | Caesarean section with multiple complication diagnoses, duration of pregnancy up to 25 weeks or with intrauterine therapy |
| **O01C** (V 1.0, V 2.0, V3.0, V4.0, V5.0) | Césarienne avec plusieurs diagnostics de complication, durée de la grossesse > 33 semaines complètes, sans thérapie intra-utérine ou avec diagnostic de complication, de 26 à 33 semaines ou avec diagnostic complexe ou jusqu'à 33 semaines ou avec diagnostic complexe, avec CC extrêmement sévères | Caesarean section with multiple complication diagnoses, duration of pregnancy > 33 completed weeks, without intrauterine therapy or with complication diagnosis, from 26 to 33 weeks or with complex diagnosis or up to 33 weeks or with complex diagnosis, with extremely severe CC |
| **O01C** (V6.0, V7.0) | Césarienne secondaire avec plusieurs diagnostics de complication ou procédure complexe, ou jusqu'à 33 semaines de grossesse ou diagnostic complexe ou diagnostic de complication et grossesse de 26 à 33 semaines ou diagnostic complexe | Secondary caesarean section with multiple complication diagnoses or complex procedure, or up to 33 weeks of pregnancy or complex diagnosis or complication diagnosis and 26-33 weeks of pregnancy or complex diagnosis |
| **O01C** (V8.0, V9.0 | Césarienne avec plusieurs diagnostics de complication, grossesse de 26 à 33 semaines ou CC extrêmement sévères ou diagnostic complexe ou proc. de complication, grossesse de jusqu'à 33 semaines ou diagnostic complexe et CC extrêmement sévères ou grossesse de jusqu'a 25 semaines et diagnostic de complication | Caesarean section with multiple complication diagnoses, pregnancy of 26-33 weeks or extremely severe CC or complex diagnosis or complication procedure, pregnancy of up to 33 weeks or complex diagnosis and extremely severe CC or pregnancy of up to 25 weeks and complication diagnosis |
| **O01D** (V 1.0, V 2.0, V3.0, V4.0, V5.0) | Césarienne avec plusieurs diagnostics de complication, durée de la grossesse > 33 semaines complètes, sans thérapie intra-utérine ou avec diagnostic de complication, de 26 à 33 semaines ou avec diagnostic complexe ou jusqu'à 33 semaines ou avec diagnostic complexe, sans CC extrêmement sévères | Caesarean section with multiple complication diagnoses, duration of pregnancy > 33 completed weeks, without intrauterine therapy or with complication diagnosis, 26 to 33 weeks or with complex diagnosis or up to 33 weeks or with complex diagnosis, without extremely severe CC |
| **O01D** (V6.0, V7.0) | Césarienne secondaire avec diagnostic de complication, durée de la grossesse plus de 33 semaines complètes | Secondary caesarean section with diagnosis of complication, duration of pregnancy over 33 completed weeks |
| **O01D** (V8.0, V9.0 | Césarienne secondaire avec plusieurs diagnostics de complication ou procédure complexe ou durée de la grossesse jusqu'à 33 semaines ou diagnostic complexe ou diagnostic de complication et grossesse de 26 à 33 semaines ou diagnostic complexe | Secondary caesarean section with multiple complication diagnoses or complex procedure or duration of pregnancy up to 33 weeks or complex diagnosis or complication diagnosis and pregnancy from 26 to 33 weeks or complex diagnosis |
| **O01E** (V 1.0, V 2.0, V3.0, V4.0, V5.0) | Césarienne avec diagnostic de complication, durée de la grossesse plus de 33 semaines complètes, sans diagnostic complexe | Caesarean section with complication diagnosis, duration of pregnancy over 33 completed weeks, without complex diagnosis |
| **O01E** (V6.0, V7.0) | Césarienne avec plusieurs diagnostics de complication ou procédure complexe, ou jusqu'à 33 semaines de grossesse ou diagnostic complexe, ou diagnostic de complication et grossesse de 26 à 33 semaines ou diagnostic complexe, ou césarienne secondaire | Caesarean section with multiple complication diagnoses or complex procedure, or up to 33 weeks of pregnancy or complex diagnosis, or complication diagnosis and 26-33 weeks of pregnancy or complex diagnosis, or secondary caesarean section |
| **O01E** (V8.0, V9.0) | Césarienne secondaire avec diagnostic de complication, durée de la grossesse de plus de 33 semaines complètes | Secondary caesarean section with complication diagnosis, pregnancy duration over 33 completed weeks |
| **O01F** (V 1.0, V 2.0, V3.0, V4.0, V5.0) | Césarienne sans diagnostic de complication, durée de la grossesse plus de 33 semaines complètes, sans diagnostic complexe | Caesarean section without diagnosis of complication, duration of pregnancy over 33 completed weeks, without complex diagnosis |
| **O01F** (V6.0 V7.0) | Césarienne avec diagnostic de complication, durée de la grossesse plus de 33 semaines complètes | Caesarean section with diagnosis of complication, duration of pregnancy more than 33 completed weeks |
| **O01F** (V8.0, V9.0 | Césarienne avec plusieurs diagnostics de complication ou procédure complexe ou grossesse jusqu'à 33 semaines ou diagnostic complexe, ou diagnostic de complication et grossesse de 26 à 33 semaines ou diagnostic complexe ou césarienne secondaire | Caesarean section with multiple complication diagnoses or complex procedure or pregnancy up to 33 weeks or complex diagnosis, or complication diagnosis and pregnancy 26-33 weeks or complex diagnosis or secondary caesarean section |
| **O01G** (V6.0, V7.0) | Césarienne, durée de la grossesse > 33 semaines complètes | Caesarean section, duration of pregnancy > 33 completed weeks |
| **O01G** (V8.0, V9.0) | Césarienne avec diagnostic de complication, durée de la grossesse plus de 33 semaines complètes | Caesarean section with complication diagnosis, duration of pregnancy > 33 completed weeks |
| **O01H** (V7.0, V8.0, V9.0) | Césarienne, durée de la grossesse plus de 33 semaines complètes | Caesarean section, duration of pregnancy > 33 completed weeks |
| **O02A** (V 1.0, V 2.0, V3.0, V4.0) | Accouchement par voie basse avec procédure opératoire de complication, durée de la grossesse jusqu'à 33 semaines complètes ou avec thérapie intra-utérine | Vaginal delivery with operative complication procedure, duration of pregnancy up to 33 completed weeks or with intrauterine therapy |
| **O02A** (V5.0, V6.0, V7.0) | Accouchement par voie basse avec procédure opératoire de complication, avec thérapie intra-utérine ou traitement complexe de soins intensifs > 119 points ou procédure de complication ou procédure complexe | Vaginal delivery with operative complication procedure, with intrauterine therapy or complex intensive care treatment > 119 points or complication procedure or complex procedure |
| **O02A** (V8.0) | Accouchement par voie basse avec intervention coûteuse, ou procédure particulière avec diagnostic particulier, avec procédure de complication ou procédure complexe | Delivery by vaginal route with costly intervention, or special procedure with special diagnosis, with complication procedure or complex procedure |
| **O02B** (V 1.0, V 2.0, V3.0, V4.0) | Accouchement par voie basse avec procédure opératoire de complication, durée de la grossesse plus de 33 semaines complètes, sans thérapie intra-utérine | Vaginal delivery with operative complication procedure, duration of pregnancy more than 33 completed weeks, without intrauterine therapy |
| **O02B** (V5.0, V6.0, V7.0) | Accouchement par voie basse avec procédure opératoire de complication | Vaginal delivery with operative complication procedure |
| **O02B** (V8.0) | Accouchement par voie basse avec intervention coûteuse, ou procédure particulière avec diagnostic particulier | Vaginal delivery with costly intervention, or special procedure with special diagnosis |
| **O60A** (V 1.0, V 2.0, V3.0) | Accouchement par voie basse avec plusieurs diagnostics de complication, au moins une complication sévère, durée de la grossesse jusqu’à 33 semaines complètes ou avec procédure de complication | Vaginal delivery with several complication diagnoses, at least one severe complication, duration of pregnancy up to 33 completed weeks or with complication procedure |
| **060A** (V 4.0, V5.0, V6.0, V7.0) | Accouchement par voie basse avec plusieurs diagnostics de complication, au moins une complication sévère, durée de la grossesse jusqu’à 33 semaines complètes ou avec procédure de complication ou thromboembolie pendant la période de gestation | Vaginal delivery with several diagnoses of complication, at least one severe complication, duration of pregnancy up to 33 completed weeks or with complication procedure or thromboembolism during the gestation period |
| **O60A** (V8.0) | Accouchement par voie basse avec plusieurs diagnostics de complication, au moins une complication sévère, ou thromboembolie pendant la période de gestation, durée de la grossesse jusqu'à 33 semaines complètes | Vaginal delivery with several diagnoses of complications, at least one severe complication, or thromboembolism during the gestation period, duration of pregnancy up to 33 completed weeks |
| **O60A** (V9.0) | Accouchement par voie basse avec plusieurs diagnostics de complication, durée de la grossesse jusqu'à 33 semaines complètes ou traitement particulier | Vaginal delivery with several diagnoses of complications, duration of pregnancy up to 33 completed weeks or special treatment |
| **O60B** (V 1.0, V 2.0) | Accouchement par voie basse avec plusieurs diagnostics de complication, au moins une complication sévère, durée de la grossesse plus de 33 semaines complètes, sans procédure de complication ou thromboembolie pendant la période de gestation avec procédure opératoire | Vaginal delivery with several diagnoses of complications, at least one severe complication, duration of pregnancy more than 33 completed weeks, without complication procedure or thromboembolism during the gestation period with operative procedure |
| **060B** (V3.0) | Accouchement par voie basse avec plusieurs diagnostics de complication, au moins une complication sévère, durée de la grossesse plus de 33 semaines complètes, sans procédure de complication ou thromboembolie pendant la période de gestation sans procédure opératoire | Vaginal delivery with several diagnoses of complications, at least one severe complication, duration of pregnancy more than 33 completed weeks, without complication procedure or thromboembolism during the gestation period without operative procedure |
| **060B** (V4.0, V5.0) | Accouchement par voie basse avec plusieurs diagnostics de complication, au moins une complication sévère, durée de la grossesse plus de 33 semaines complètes, sans procédure de complication ou thromboembolie pendant la période de gestation | Vaginal delivery with several diagnoses of complications, at least one severe complication, duration of pregnancy more than 33 completed weeks, without complication procedure or thromboembolism during the gestation period |
| **O60B** (V6.0, V7.0) | Accouchement par voie basse avec plusieurs diagnostics de complication, au moins une complication sévère, durée de la grossesse plus de 33 semaines complètes | Vaginal delivery with several diagnoses of complications, at least one severe complication, duration of pregnancy more than 33 completed weeks |
| **O60B** (V8.0) | Accouchement par voie basse avec plusieurs diagnostics de complication, au moins une complication sévère, ou thromboembolie pendant la période de gestation, durée de la grossesse plus de 33 semaines complètes | Vaginal delivery with several diagnoses of complications, at least one severe complication, or thromboembolism during the gestation period, duration of pregnancy more than 33 full weeks |
| **O60B** (V9.0) | Accouchement par voie basse avec plusieurs diagnostics de complication, durée de la grossesse plus de 33 semaines complètes | Vaginal delivery with several diagnoses of complications, duration of pregnancy more than 33 completed weeks |
| **O60C** (V 1.0, V 2.0, V3.0, V4.0, V5.0, V6.0, V7.0) | Accouchement par voie basse avec diagnostic de complication sévère ou moyennement sévère | Vaginal delivery with diagnosis of severe or moderately severe complication |
| **O60C** (V8.0) | Accouchement par voie basse avec diagnostic de complication sévère ou moyennement sévère ou procédure particulière | Vaginal delivery with diagnosis of severe or moderately severe complication or special procedure |
| **O60C** (V9.0) | Accouchement par voie basse avec diagnostic de complication sévère ou moyennement sévère ou traitement particulier | Vaginal delivery with diagnosis of severe or moderately severe complication or special treatment |
| **O60D** (V 1.0, V 2.0, V3.0, V4.0) | Accouchement par voie basse sans diagnostic de complication | Delivery by vaginal delivery without diagnosis of a complication |
| **060D** (V5.0, V6.0, V7.0, V8.0, V9.0) | Accouchement par voie basse | Delivery by vaginal route |
| **O02Z** (V9.0) | Accouchement par voie basse avec intervention coûteuse, ou procédure particulière avec diagnostic particulier | Childbirth by vaginal delivery with costly intervention, or special procedure with special diagnosis |

Supplemental Table 2: Relevant codes to identify a preterm delivery.

|  | **Original codes** | **English translation** |
| --- | --- | --- |
| **SwissDRG Codes** | **MDC 15: Nouveaux nés** | **MDC 15: Newborns** |
| **O01A** (V 1.0, V 2.0, V3.0, V4.0, V 5.0, V6.0) | Césarienne avec plusieurs diagnostics de complication, durée de la grossesse jusqu'à 25 semaines complètes ou avec thérapie intra-utérine | Caesarean section with multiple complication diagnoses, duration of pregnancy up to 25 completed weeks or with intrauterine therapy |
| **O01A** (V7.0, V8.0, V9.0) | Césarienne et dialyse, ou thérapie intra-utérine complexe du fœtus | Caesarean section and dialysis, or complex intrauterine fetal therapy |
| **O01B** (V 1.0, V 2.0, V3.0, V4.0 | Césarienne avec plusieurs diagnostics de complication, durée de la grossesse de 26 à 33 semaines complètes, sans thérapie intra-utérine ou avec diagnostic de complication, jusqu'à 25 semaines complètes, ou thromboembolie pendant la période de gestation avec procédure opératoire | Caesarean section with several diagnoses of complication, duration of pregnancy from 26 to 33 completed weeks, without intrauterine therapy or with diagnosis of complication, up to 25 completed weeks, or thromboembolism during the gestation period with operative procedure |
| **O01B** (V5.0) | Césarienne avec plusieurs diagnostics de complication, durée de la grossesse de 26 à 33 semaines complètes, jusqu'à 25 semaines complètes, ou thromboembolie pendant la période de gestation avec procédure opératoire ou procédure complexe | Caesarean section with several diagnoses of complication, duration of pregnancy from 26 to 33 completed weeks, up to 25 completed weeks, or thromboembolism during the gestation period with operative procedure or complex procedure |
| **O01B** (V6.0, V7.0) | Césarienne avec plusieurs diagnostics de complication, durée de la grossesse de 26 à 33 semaines ou CC extrêmement sévères ou diagnostic complexe ou procédure de complication, jusqu'à 33 semaines de grossesse ou diagnostic complexe et CC extrêmement sévères, ou jusqu'à 25 semaines de grossesse et diagnostic de complication | Caesarean section with multiple complication diagnoses, duration of pregnancy 26-33 weeks or extremely severe CC or complex diagnosis or complication procedure, up to 33 weeks of pregnancy or complex diagnosis and extremely severe CC, or up to 25 weeks of pregnancy and complication diagnosis |
| **O01B** (V8.0, V9.0) | Césarienne avec plusieurs diagnostics de complication, durée de la grossesse jusqu'à 25 semaines ou avec thérapie intra-utérine | Caesarean section with multiple complication diagnoses, duration of pregnancy up to 25 weeks or with intrauterine therapy |
| **O01C** (V 1.0, V 2.0, V3.0, V4.0, V5.0) | Césarienne avec plusieurs diagnostics de complication, durée de la grossesse > 33 semaines complètes, sans thérapie intra-utérine ou avec diagnostic de complication, de 26 à 33 semaines ou avec diagnostic complexe ou jusqu'à 33 semaines ou avec diagnostic complexe, avec CC extrêmement sévères | Caesarean section with multiple complication diagnoses, duration of pregnancy > 33 completed weeks, without intrauterine therapy or with complication diagnosis, from 26 to 33 weeks or with complex diagnosis or up to 33 weeks or with complex diagnosis, with extremely severe CC |
| **O01C** (V6.0, V7.0) | Césarienne secondaire avec plusieurs diagnostics de complication ou procédure complexe, ou jusqu'à 33 semaines de grossesse ou diagnostic complexe ou diagnostic de complication et grossesse de 26 à 33 semaines ou diagnostic complexe | Secondary caesarean section with multiple complication diagnoses or complex procedure, or up to 33 weeks of pregnancy or complex diagnosis or complication diagnosis and 26-33 weeks of pregnancy or complex diagnosis |
| **O01C** (V8.0, V9.0 | Césarienne avec plusieurs diagnostics de complication, grossesse de 26 à 33 semaines ou CC extrêmement sévères ou diagnostic complexe ou proc. de complication, grossesse de jusqu'à 33 semaines ou diagnostic complexe et CC extrêmement sévères ou grossesse de jusqu'a 25 semaines et diagnostic de complication | Caesarean section with multiple complication diagnoses, pregnancy of 26-33 weeks or extremely severe CC or complex diagnosis or complication procedure, pregnancy of up to 33 weeks or complex diagnosis and extremely severe CC or pregnancy of up to 25 weeks and complication diagnosis |
| **O01D** (V 1.0, V 2.0, V3.0, V4.0, V5.0) | Césarienne avec plusieurs diagnostics de complication, durée de la grossesse > 33 semaines complètes, sans thérapie intra-utérine ou avec diagnostic de complication, de 26 à 33 semaines ou avec diagnostic complexe ou jusqu'à 33 semaines ou avec diagnostic complexe, sans CC extrêmement sévères | Caesarean section with multiple complication diagnoses, duration of pregnancy > 33 completed weeks, without intrauterine therapy or with complication diagnosis, 26 to 33 weeks or with complex diagnosis or up to 33 weeks or with complex diagnosis, without extremely severe CC |
| **O01D** (V6.0, V7.0) | Césarienne secondaire avec diagnostic de complication, durée de la grossesse plus de 33 semaines complètes | Secondary caesarean section with complication diagnosis, duration of pregnancy more than 33 completed weeks |
| **O01D** (V8.0, V9.0 | Césarienne secondaire avec plusieurs diagnostics de complication ou procédure complexe ou durée de la grossesse jusqu'à 33 semaines ou diagnostic complexe ou diagnostic de complication et grossesse de 26 à 33 semaines ou diagnostic complexe | Secondary caesarean section with multiple complication diagnoses or complex procedure or duration of pregnancy up to 33 weeks or complex diagnosis or complication diagnosis and pregnancy from 26 to 33 weeks or complex diagnosis |
| **O01E** (V 1.0, V 2.0, V3.0, V4.0, V5.0) | Césarienne avec diagnostic de complication, durée de la grossesse plus de 33 semaines complètes, sans diagnostic complexe | Caesarean section with complication diagnosis, duration of pregnancy more than 33 completed weeks, without complex diagnosis |
| **O01E** (V6.0, V7.0) | Césarienne avec plusieurs diagnostics de complication ou procédure complexe, ou jusqu'à 33 semaines de grossesse ou diagnostic complexe, ou diagnostic de complication et grossesse de 26 à 33 semaines ou diagnostic complexe, ou césarienne secondaire | Caesarean section with multiple complication diagnoses or complex procedure, or up to 33 weeks of pregnancy or complex diagnosis, or complication diagnosis and 26-33 weeks of pregnancy or complex diagnosis, or secondary caesarean section |
| **O01E** (V8.0, V9.0) | Césarienne secondaire avec diagnostic de complication, durée de la grossesse de plus de 33 semaines complètes | Secondary caesarean section with complication diagnosis, pregnancy duration over 33 completed weeks |
| **O01F** (V 1.0, V 2.0, V3.0, V4.0, V5.0) | Césarienne sans diagnostic de complication, durée de la grossesse plus de 33 semaines complètes, sans diagnostic complexe | Caesarean section without diagnosis of complication, duration of pregnancy over 33 completed weeks, without complex diagnosis |
| **O01F** (V6.0 V7.0) | Césarienne avec diagnostic de complication, durée de la grossesse plus de 33 semaines complètes | Caesarean section with diagnosis of complication, duration of pregnancy more than 33 completed weeks |
| **O01F** (V8.0, V9.0 | Césarienne avec plusieurs diagnostics de complication ou procédure complexe ou grossesse jusqu'à 33 semaines ou diagnostic complexe, ou diagnostic de complication et grossesse de 26 à 33 semaines ou diagnostic complexe ou césarienne secondaire | Caesarean section with multiple complication diagnoses or complex procedure or pregnancy up to 33 weeks or complex diagnosis, or complication diagnosis and pregnancy 26-33 weeks or complex diagnosis or secondary caesarean section |
| **O60A** (V 1.0, V 2.0, V3.0) | Accouchement par voie basse avec plusieurs diagnostics de complication, au moins une complication sévère, durée de la grossesse jusqu’à 33 semaines complètes ou avec procédure de complication | vaginal delivery with multiple complication diagnoses, at least one severe complication, duration of pregnancy up to 33 completed weeks or with complication procedure |
| **O60A** (V 4.0, V5.0, V6.0, V7.0) | Accouchement par voie basse avec plusieurs diagnostics de complication, au moins une complication sévère, durée de la grossesse jusqu’à 33 semaines complètes ou avec procédure de complication ou thromboembolie pendant la période de gestation | vaginal delivery with several diagnoses of complications, at least one severe complication, duration of pregnancy up to 33 completed weeks or with complication procedure or thromboembolism during the gestation period |
| **O60A** (V8.0) | Accouchement par voie basse avec plusieurs diagnostics de complication, au moins une complication sévère, ou thromboembolie pendant la période de gestation, durée de la grossesse jusqu'à 33 semaines complètes | vaginal delivery with several diagnoses of complication, at least one severe complication, or thromboembolism during the gestation period, duration of pregnancy up to 33 completed weeks |
| **O60A** (V9.0) | Accouchement par voie basse avec plusieurs diagnostics de complication, durée de la grossesse jusqu'à 33 semaines complètes ou traitement particulier | vaginal delivery with several diagnoses of complications, duration of pregnancy up to 33 completed weeks or special treatment |

*Supplemental Table 3:* Prevalence of exposure to insulin, to blood glucose lowering drugs, or to both insulin and blood glucose lowering drugs, among continuers, switchers, and discontinuers during the pre-pregnancy period, overall and per year between 2012 and 2019.

|  | 2012 | 2013 | 2014 | 2015 | 2016 | 2017 | 2018 | 2019 | 2012-2019 |
| --- | --- | --- | --- | --- | --- | --- | --- | --- | --- |
| **Total pregnancies, n** | **10,639** | **11,484** | **12,306** | **12,917** | **13,780** | **13,803** | **14,525** | **14,644** | **104'098** |
| **Pregnancies exposed to an ADM (A10) during the pre-pregnancy period (N/10000 (95% CI))** | **29.1 (19.8-41.1)** | **14.8 (7.9-23.2)** | **27.6 (19.1-38.6)** | **34.1 (24.8-45.7)** | **36.3 (26.9-47.8)** | **41.3 (31.3-53.5)** | **48.9 (38.2-61.6)** | **51.9 (40.9-64.9)** | **36.5 (32.9-40.4)** |
| Exposed to insulin (A10A) | 24.4 (16.0-35.8) | 13.1 (7.3-21.5) | 21.9 (14.5-31.9) | 23.2 (15.7-33.1) | 26.1(18.3-36.2) | 24.6 (17.1-34.4) | 18.6 (12.2-27.0) | 23.2 (16.1-32.4) | 22.0 (19.2-25.0) |
| Exposed to a blood glucose lowering drug (A10B) | 2.8 (1.0-9.6) | 1.7 (0.2-6.3) | 4.9 (1.8-10.6) | 10.8 (5.9-18.2) | 9.4 (5.0-16.1) | 13.8 (8.3-21.5) | 27.5 (19.7-37.5) | 26.6 (19.0-36.4) | 13.1 (11.0-15.5) |
| Exposed to both (A10A + A10B) | 1.9 (0.2-6.8) | 0.0 (0.0-3.2) | 0.8 (0.02-4.5) | 0.0 (0.0-2.9) | 0.7 (0.02-4.0) | 2.9 (0.8-7.4) | 2.8 (0.8-7.0) | 2.0 (0.4-6.0) | 1.4 (0.8-2.4) |
| **Pregnancies exposed to an ADM (A10) during the pre-pregnancy period and in or after T2 (Pregestational diabetes mellitus group)** | **24.4 (16.0-35.8)** | **11.3 (6.0-19.4)** | **22.8 (15.1-32.9)** | **27.1 (18.9-37.7)** | **27.6 (19.5-37.8)** | **31.2 (22.6-41.9)** | **28.9 (20.8-39.1)** | **30.7 (22.4-41.1)** | **25.9 (22.9-29.2)** |
| **Continuers^1^** | 23.5 (15.2-34.7) | 11.3 (6.0-19.4) | 21.1 (13.8-30.9) | 22.5 (15.0-32.2) | 23.2 (15.9-32.8) | 26.8 (18.9-36.9) | 21.4 (14.5-30.3) | 25.3 (17.8-34.8) | 22.1 (19.3-25.1) |
| Exposed to insulin^2^ (A10A) | 21.6 (13.7-32.4) | 11.3 (6.0-19.4) | 20.3 (13.2-30.0) | 21.7 (14.4-31.3) | 22.5 (15.3-31.9) | 22.5 (15.3-31.9) | 15.8 (10.0-23.8) | 19.8 (13.3-28.4) | 19.5 (16.9-22.4) |
| Exposed to a blood glucose lowering drug^3^ (A10B) | 0.0 (0.0-3.5) | 0.0 (0.0-3.2) | 0.0 (0.0-3.0) | 0.8 (0.02 -4.3) | 0.0 (0.0-2.7) | 1.4 (0.2-5.2) | 2.8 (0.8-7.0) | 3.4 (1.1-8.0) | 1.2 (0.7-2.1) |
| Exposed to both in the pre-pregnancy period and in or after T2 (A10A + A10B) | 0.0 (0.0-3.5) | 0.0 (0.0-3.2) | 0.0 (0.0-3.0) | 0.0 (0.0-2.9) | 0.0 (0.0-2.7) | 0.0 (0.0-2.7) | 0.7 (0.02-3.8) | 0.7 (0.02-3.8) | 0.2 (0.02-0.7) |
| Exposed to both in the pre-pregnancy period and to insulin only in or after T2 (A10A + A10B) | 1.9 (0.2-6.8) | 0.0 (0.0-3.2) | 0.8 (0.02-4.5) | 0.0 (0.0-2.9) | 0.7 (0.02-4.0) | 2.9 (0.8-7.4) | 2.1 (0.4-6.0) | 1.4 (0.2-4.9) | 1.2 (0.7-2.1) |
| Exposed to both in the pre-pregnancy period and to blood glucose lowering drugs only in or after T2 (A10A + A10B) | 0.0 (0.0-3.5) | 0.0 (0.0-3.2) | 0.0 (0.0-3.0) | 0.0 (0.0-2.9) | 0.0 (0.0-2.7) | 0.0 (0.0-2.7) | 0.0 (0.0-2.5) | 0.0 (0.0-2.5) | 0.0 (0.0-3.5) |
| **Switchers^4^** | 0.9 (0.02-5.2) | *0.0 (0.0-3.2)* | 1.6 (0.2-5.9) | 4.6 (1.7-10.1) | 4.4 (1.6-9.5) | 4.3 (1.6-9.5) | 7.6 (3.8-13.5) | 5.5 (2.4-10.8) | 3.8 (2.7-5.2) |
| Blood glucose lowering drug to insulin^5^ (A10B) | 0.9 (0.02-5.2) | 0.0 (0.0-3.2) | 1.6 (0.2 -5.9) | 4.6 (1.7-10.1) | 4.4 (1.6-9.5) | 4.3 (1.6-9.5) | 7.6 (3.8-13.5) | 5.5 (2.4-10.8) | 3.8 (2.7-5.2) |
| **Pregnancies exposed to an ADM (A10) during the pre-pregnancy period and unexposed in or after T2 (Discontinuers)** | 4.7 (1.5-11.0) | 3.5 (0.9-8.9) | 4.9 (4.86-4.94) | 7.7 (3.7-14.2) | 8.7 (0.5-15.2) | 10.1 (5.5-17.0) | 20.0 (13.4-28.7) | 21.2 (14.4-30.3) | 10.7 (5.2-18.9) |
| Insulin discontinuers^6^ (A10A) | 2.8 (0.6-8.2) | 1.7 (0.2-6.3) | 1.6 (0.2 -5.9) | 1.5 (0.2 -5.6) | 3.6 (1.2-8.5) | 2.2 (0.4-6.4) | 2.8 (0.8-7.0) | 3.4 (1.1-7.9) | 2.5 (1.6-3.6) |
| Blood glucose lowering drugs discontinuers^7^ (A10B) | 1.9 (0.02-6.8) | 1.7 (0.2 -6.3) | 3.3 (0.9-8.3) | 5.4 (2.2-11.2) | 5.1 (2.0-10.5) | 8.0 (4.0-14.3) | 17.2 (11.1-25.4) | 17.8 (11.6-26.0) | 8.1 (6.5-10.1) |

^1^ Pregnancies exposed to an ADM during the pre-pregnancy period and to the same ADM in or after T2; ^2^Pregnancies exposed to insulin in the pre-pregnancy period and also exposed to insulin in or after T2; ^3^Pregnancies exposed to a blood glucose lowering drug in the pre-pregnancy period and also exposed to a blood glucose lowering drug in or after T2; ^4^ Pregnancies exposed to an ADM during the pre-pregnancy period and exposed to a different ADM in or after T2; ^5^Pregnancies exposed to a blood glucose lowering drug in the pre-pregnancy period and exposed to insulin in or after T2; ^6^Pregnancies exposed to insulin in the pre-pregnancy period and unexposed to any ADM in or after T2; ^7^Pregnancies exposed to a blood glucose lowering drug in the pre-pregnancy period and unexposed to any ADM in or after T2.

Supplemental Table 4: Proportion of different insulin and blood glucose lowering drugs prescriptions within each group of pregestational diabetes mellitus (continuers, switchers) during the pre-pregnancy period, overall and per year, between 2012 and 2019.

|  | 2012 | 2013 | 2014 | 2015 | 2016 | 2017 | | 2018 | | 2019 | 2012-2019 |
| --- | --- | --- | --- | --- | --- | --- | --- | --- | --- | --- | --- |
| **Total pregnancies (N)** | **10,639** | **11,484** | **12,306** | **12,917** | **13,780** | **13,803** | | **14,525** | | **14,644** | **104'098** |
| **Continuers** | | | | | | | | | | | |
| ***Insulin continuers^3^*** | | | | | | | | | | | |
| Total insulin prescriptions (A10A) (N, (%, 95%CI )) | 135.0 (100.0) | 61.0 (100.0) | 142.0 (100.0) | 135.0 (100.0) | 187.0 (100.0) | 179.0 (100.0) | | 124.0 (100.0) | | 223.0 (100.0) | 1186.0 (100.0) |
| Insulin aspart *(A10AB05)* | 64.0 (47.4, 38.8-56.2) | 21.0 (34.4, 22.7-47.7) | 43.0 (30.3, 22.9-38.5) | 66.0 (48.9, 40.2-57.6) | 71.0 (38.0, 31.0-45.3) | 72.0 (40.2, 33.0-47.8) | | 47.0 (37.9, 29.3-47.1) | | 113.0 (50.7, 43.9-57.4) | 497.0 (42.3, 39.1-44.8) |
| Insulin lispro *(A10AB04, A10AD04)* | 45.0 (33.3, 24.5-42.0) | 16.0 (26.2, 15.8-39.1) | 39.0 (27.5, 20.3-35.6) | 30.0 (22.2, 15.5-30.2) | 53.0 (28.3, 22.0-35.4) | 22.0 (12.3, 7.9-18.0) | | 18.0 (14.5, 8.8-22.0) | | 23.0 (10.3, 6.7-15.1) | 246.0 (20.9, 18.5-23.2) |
| Insulin detemir *(A10AE05)* | 4.0 (3.0, 0.8-7.4) | 6.0 (9.8, 3.7-20.2) | 16.0 (11.3, 6.6-17.7) | 17.0 (12.6, 7.5-19.4) | 32.0 (17.1, 12.0-23.3) | 41.0 (22.9, 17.0-29.8) | | 19.0 (15.3, 9.5-22.9) | | 38.0 (17.0, 12.3-22.6) | 173.0 (14.7, 12.6-16.7) |
| Insulin glargine *(A10AE04)* | 12.0 (8.9, 4.7-15.0) | 5.0 (8.2, 2.7-18.1) | 24.0 (16.9, 11.1-24.1) | 11.0 (8.1, 4.1-14.1) | 19.0 (10.2, 6.2-15.4) | 24.0 (13.4, 8.8-19.3) | | 6.0 (4.8, 1.8-10.2) | | 34.0 (15.2, 10.8-20.6) | 135.0 (11.5, 9.6-13.3) |
| Human insulin *(A10AB01, A10AC01)* | 10 (7.4, 3.6-13.2) | 7.0 (11.5, 4.7-22.2) | 13.0 (9.2, 5.0-15.1) | 5.0 (3.7, 1.2-8.4) | 7.0 (3.7, 1.5-7.6) | 1.0 (0.6, 0.01-3.1) | | 13.0 (10.5, 5.7-17.3) | | 3.0 (1.3, 0.3-3.9) | 59.0 (5.0, 3.8-6.4) |
| Insulin degludec *(A10AE06)* | 0.0 (0.0, 0.0-2.7) | 0.0 (0.0, 0.0-5.9) | 6.0 (4.2, 1.6-9.0) | 4.0 (3.0, 0.8-7.4) | 5.0 (2.7, 0.9-6.1) | 16.0 (8.9, 5.2-14.1) | | 12.0 (9.7, 5.1-16.3) | | 3.0 (1.3, 0.3-3.9) | 46.0 (3.9, 2.9-5.1) |
| Insulin glulisine *(A10AB06)* | 0.0 (0.0, 0.0-2.7) | 6.0 (9.8, 3.7-20.2) | 1.0 (0.7, 0.2-3.9) | 2.0 (1.5, 0.2-5.2) | 0.0 (0.0, 0.0-2.0) | 3.0 (1.7, 0.3-4.8) | | 9.0 (7.3, 3.4-13.3) | | 9.0 (4.0, 1.9-7.5) | 30.0 (2.6, 1.7-3.6) |
| ***Blood glucose lowering drugs*** ***continuers^4^*** | | | | | | | | | | | |
| Total blood glucose lowering drugs prescriptions (A10B) (N, (%)) | 0.0 (0.0, 0.0-2.7) | 0.0 (0.0, 0.0-5.9) | 0.0 (0.0-2.6) | 1.0 (100.0) | 0.0 (0.0, 0.0-2.0) | | 8.0 (100.0) | | 32.0 (100.0) | 22.0 (100.0) | 62.0 (100.0) |
| Metformin | 0.0 (0.0, 0.0-2.7) | 0.0 (0.0, 0.0-5.9) | 0.0 (0.0-2.6) | 1.0 (100.0, 25.0-100.0) | 0.0 (0.0, 0.0-2.0) | | 7.0 (87.5, 47.3-99.7) | | 13.0 (40.6, 23.7-59.4) | 21.0 (95.5, 77.2-99.9) | 41.0 (66.1, 60.0-77.7) |
| Glucagon-like peptide 1 (GLP-1) receptor agonist (Dulaglutide, Liraglutide) (A10BJ) | 0.0 (0.0, 0.0-2.7) | 0.0 (0.0, 0.0-5.9) | 0.0 (0.0-2.6) | 0.0 (0.0, 0.0-97.5) | 0.0 (0.0, 0.0-2.0) | | 0.0 (0.0, 0.0-36.9) | | 13.0 (40.6, 23.7-59.4) | 0.0 (0.0, 0.0-15.4) | 13.0 (21.0, 11.7-33.2) |
| Sodium-glucose cotransporter-2 (SGLT2) inhibitors (A10BK) | 0.0 (0.0, 0.0-2.7) | 0.0 (0.0, 0.0-5.9) | 0.0 (0.0-2.6) | 0.0 (0.0, 0.0-97.5) | 0.0 (0.0, 0.0-2.0) | | 1.0 (12.5, 0.3-52.7) | | 4.0 (12.5, 3.5-29.0) | 0.0 (0.0, 0.0-15.4) | 5.0 (8.1, 2.7-17.8) |
| Dipeptidyl peptidase 4 (DPP-4) inhibitors (A10BH) | 0.0 (0.0, 0.0-2.7) | 0.0 (0.0, 0.0-5.9) | 0.0 (0.0-2.6) | 0.0 (0.0, 0.0-97.5) | 0.0 (0.0, 0.0-2.0) | | 0.0 (0.0, 0.0-36.9) | | 2.0 (6.2, 0.8-20.8) | 0.0 (0.0, 0.0-15.4) | 2.0 (3.2, 0.4-11.2) |
| Sulfonylureas (A10BB) | 0.0 (0.0, 0.0-2.7) | 0.0 (0.0, 0.0-5.9) | 0.0 (0.0-2.6) | 0.0 (0.0, 0.0-97.5) | 0.0 (0.0, 0.0-2.0) | | 0.0 (0.0, 0.0-36.9) | | 0.0 (0.0, 0.0-10.9) | 1.0 (4.5, 0.0-22.8) | 1.0 (1.6, 0.04-8.7) |
| **Switchers** | | | | | | | | | | | |
| ***Blood glucose lowering drugs*** ***switchers^5^*** | | | | | | | | | | | |
| Total blood glucose lowering drugs prescriptions (N, (%)) | 1.0 (100.0, 25.0-100.0) | 0.0 (0.0) | 6.0 (100.0) | 29.0 (100.0) | 26.0 (100.0) | 20.0 (100.0) | | 21.0 (100.0) | | 30.0 (100.0) | 133.0 (100.0) |
| Metformin | 0.0 (0.0, 0.0-97.5) | 0.0 (0.0) | 3.0 (50.0, 11.8-88.2) | 4.0 (13.8, 3.9-31.7) | 2.0 (7.7, 0.9-25.1) | 4.0 (20.0, 5.7-43.7) | | 11.0 (52.4, 29.8-74.3) | | 19.0 (63.3, 43.9-80.1) | 43.0 (32.3, 24.5-41.0) |
| DPP-4 inhibitors | 1.0 (100.0, 25.0-100.0) | 0.0 (0.0) | 0.0 (0.0, 0.0-45.9) | 9.0 (31.0, 15.3-50.8) | 7.0 (26.9, 11.6-47.8) | 12.0 (60.0, 36.1-80.9) | | 0.0 (0.0, 0.0-16.1) | | 1.0 (3.3, 0.0-17.2) | 30.0 (22.6, 15.8-30.6) |
| GLP-1 receptor agonist (Liraglutide, semaglutide) | 0.0 (0.0, 0.0-97.5) | 0.0 (0.0) | 0.0 (0.0, 0.0-45.9) | 0.0 (0.0, 0.0-11.9) | 9.0 (34.6, 17.2-55.7) | 1.0 (5.0, 12.7-24.9) | | 4.0 (19.0, 5.4-41.9) | | 10.0 (33.3, 17.3-52.8) | 24.0 (18.0, 11.9-25.6) |
| Sulfonylureas | 0.0 (0.0, 0.0-97.5) | 0.0 (0.0) | 3.0 (50.0, 11.2-88.2) | 6.0 (20.7, 8.0-39.7) | 2.0 (7.7, 0.9-25.1) | 0.0 (0.0, 0.0-16.8) | | 3.0 (14.3, 3.0-36.3) | | 0.0 (0.0, 0.0-11.6) | 14.0 (10.5, 5.9-17.0) |
| SGLT2 inhibitors (empagliflozine, dapagliflozine) | 0.0 (0.0, 0.0-97.5) | 0.0 (0.0) | 0.0 (0.0, 0.0-45.9) | 0.0 (0.0, 0.0-11.9) | 6.0 (23.1, 0.9-43.6) | 3.0 (15.0, 3.2-37.9) | | 2.0 (9.5, 1.2-30.4) | | 0.0 (0.0, 0.0-11.6) | 11.0 (8.3, 4.2-14.3) |
| Other blood glucose lowering drugs used in diabetes (only repaglinide) (A10BX) | 0.0 (0.0, 0.0-97.5) | 0.0 (0.0) | 0.0 (0.0, 0.0-45.9) | 10.0 (34.5, 17.9-54.3) | 0.0 (0.0, 0.0-13.2) | 0.0 (0.0, 0.0-16.8) | | 0.0 (0.0, 0.0-1.6) | | 0.0 (0.0, 0.0-11.6) | 10.0 (7.5, 3.7-13.4) |
| Thiazolidinediones (A10BG) | 0.0 (0.0, 0.0-97.5) | 0.0 (0.0) | 0.0 (0.0, 0.0-45.9) | 0.0 (0.0, 0.0-11.9) | 0.0 (0.0, 0.0-13.2) | 0.0 (0.0, 0.0-16.8) | | 1.0 (4.8, 0.1-23.8) | | 0.0 (0.0, 0.0-11.6) | 1.0 (0.8, 0.02-4.1) |

*Supplemental Table 5: ATC codes of medication used in the treatment of infertility (with a Swissmedic number available in Switzerland in October 2022)^*^.*

| ATC code | Substance |
| --- | --- |
| G0GA | Gonadotropins |
| *G03GA01* | *Chorionic gonadotrophin* |
| *G03GA02* | *Human menopausal gonadotrophin* |
| *G03GA04* | *Urofollitropin* |
| *G03GA05* | *Follitropin alfa* |
| *G03GA06* | *Follitropin beta* |
| *G03GA07* | *Lutropin alfa* |
| *G03GA08* | *Chorionogonadotropin alfa* |
| L02AE | Gonadotropin releasing hormones analogues |
| *L02AE02* | *Leuroprorelin* |
| *L02AE04* | *Triptorelin* |
| L02B | Aromatase inhibitors |
| *L02BG03* | *Anastrozole* |
| *L02BG04* | *Létrozole* |

*Medications in the MAMA cohort were coded through Swissmedic codes. Certain medications available in Switzerland during the observation period (2012-2019) may no longer be available at the time of analysis (October 2022) and thus will no longer have a Swissmedic number. Some of these medications included clomifene (ATC G03GB02), which is known for its use in ovulation induction among women with PCOS [29].

Supplemental Table 6: Proportion of different insulin and blood glucose lowering drugs prescriptions among discontinuers overall and per year, between 2012 and 2019.

| **Discontinuers** | | | | | | | | | |
| --- | --- | --- | --- | --- | --- | --- | --- | --- | --- |
| ***Insulin discontinuers*** | | | | | | | | | |
|  | 2012 | 2013 | 2014 | 2015 | 2016 | 2017 | 2018 | 2019 | 2012-2019 |
| Total insulin prescriptions (N, (%)) | 3.0 (100.0) | 2.0 (100.0) | 5.0 (100.0) | 3.0 (100.0) | 5.0 (100.0) | 3.0 (100.0) | 4.0 (100.0) | 5.0 (100.0) | 30 (100.0) |
| Human insulin *(A10AB01, A10AC01)* | 2.0 (66.7, 9.4-99.2) | 1.0 (50.0, 12.6-98.7) | 1.0 (20.0, 5.1-71.6) | 2.0 (66.7, 9.4-99.2) | 3.0 (60.0, 14.7-94.7) | *0.0 (0.0, 0.0-70.8)* | 1.0 (25.0, 0.6-80.6) | *1.0 (20.0, 0.5-71.6)* | *11.0 (36.7, 19.9-56.1)* |
| Insulin aspart *(A10AB05)* | *0.0 (0.0, 0.0-70.8)* | 1.0 (50.0, 12.6-98.7) | 4.0 (80.0, 28.4-99.5) | *0.0 (0.0, 0.0-70.8)* | 1.0 (20.0, 5.1-71.6) | 1.0 (33.3, 0.8-90.6) | 2.0 (50.0, 6.8-93.2) | *2.0 (40.0, 5.3-85.3)* | *11.0 (36.7, 19.9-56.1)* |
| Insulin detemir *(A10AE05)* | 1.0 (33.3, 0.8-90.6) | 0.0 (0.0, 84.2) | 0.0 (0.0, 0.0-52.2) | 1.0 (33.3, 0.8-90.6) | 1.0 (20.0, 5.1-71.6) | *0.0 (0.0, 0.0-70.8*) | *1.0 (25.0, 0.6-80.6)* | *2.0 (40.0, 5.3-85.3)* | *6.0 (20.0, 7.7-38.6)* |
| Insulin lispro *(A10AB04, A10AD04)* | 0.0 (0.0, 0.0, 0.0-70.8) | 0.0 (0.0, 84.2) | 0.0 (0.0, 0.0-52.2) | 0.0 (0.0, 0.0, 0.0-70.8) | 0.0 (0.0, 0.0-52.2) | 2.0 (66.7, 9.4-99.2) | 0.0 (0.0, 0.0-60.2) | 0.0 (0.0, 0.0-52.2) | *2.0 (6.7, 0.8-22.1)* |
| Insulin degludec *(A10AE06)* | 0.0 (0.0, 0.0-70.8) | 0.0 (0.0, 84.2) | 0.0 (0.0, 0.0-52.2) | 0.0 (0.0, 0.0, 0.0-70.8) | 0.0 (0.0, 0.0-52.2) | 0.0 (0.0, 0.0, 0.0-70.8) | 0.0 (0.0, 0.0-60.2) | 0.0 (0.0, 0.0-52.2) | *0.0 (0.0, 0.0-11.6)* |
| ***Blood glucose lowering drugs discontinuers*** | | | | | | | | | |
| Total blood glucose lowering drugs prescriptions (N, (%)) | 2.0 (100.0) | 2.0 (100.0) | 4.0 (100.0) | 17.0 (100.0) | 14.0 (100.0) | 24.0 (100.0) | 48.0 (100.0) | 60.0 (100.0) | 171.0 (100.0) |
| Metformin | 0.0 (0.0, 0.0-84.2) | 2.0 (100.0) | 2.0 (50.0, 6.8-93.2) | 12.0 (70.6, 44.0-89.7) | 13.0 (92.9, 66.1-99.8) | 24.0 (100.0) | 44.0 (91.7, 80.0-97.7) | 54.0 (90.0, 79.5-96.2) | 151.0 (88.3, 82.5-92.7) |
| GLP-1 receptor agonist (Liraglutide) | 0.0 (0.0, 0.0-84.2) | 0.0 (0.0, 84.2) | 1.0 (25.0, 6.3-80.6) | 3.0 (17.6, 3.8-43.4) | 0.0 (0.0, 0.0-23.2) | 0.0 (0.0, 0.0-14.2) | 4.0 (8.3, 2.3-20.0) | 6.0 (10.0, 3.8-20.5) | 14.0 (8.2, 4.5-13.4) |
| DPP-4 inhibitors | 2.0 (100.0, 15.8-100.0) | 0.0 (0.0, 84.2) | 0.0 (0.0, 0.0-60.2) | 0.0 (0.0, 0.0-19.5) | 1.0 (7.1, 0.2-33.9) | 0.0 (0.0, 0.0-14.2) | 0.0 (0.0, 0.0-7.4) | 0.0 (0.0, 0.0-6.0) | 3.0 (1.8, 0.4-5.0) |
| Sulfonylureas | 0.0 (0.0, 0.0-84.2) | 0.0 (0.0, 84.2) | 1.0 (25.0, 6.3-80.6) | 2.0 (11.8, 1.5-36.4) | 0.0 (0.0, 0.0-23.2) | 0.0 (0.0, 0.0-14.2) | 0.0 (0.0, 0.0-7.4) | 0.0 (0.0, 0.0-6.0) | 3.0 (1.8, 0.4-5.0) |

Supplemental Table 7: Prevalence of pregnancies exposed to any ADM, to insulin, to a blood glucose lowering drug, or to both insulin and a blood glucose lowering drug, in the gestational diabetes mellitus group, overall and per year between 2012 and 2019.

|  | 2012 | 2013 | 2014 | 2015 | 2016 | 2017 | 2018 | 2019 | 2012-2019 |
| --- | --- | --- | --- | --- | --- | --- | --- | --- | --- |
| **Total pregnancies** | **10,639** | **11,484** | **12,306** | **12,917** | **13,780** | **13,803** | **14,525** | **14,644** | **104'098** |
| **Pregnancies exposed to an ADM (A10) for the first time in or after T2 (/10000, 95%CI)** | 141.9 (120.3-166.3) | 195.9 (171.4 -223.0) | 215.3 (190.4-242.6) | 245.4 (219.4-273.6**)** | 261.2 (235.3-289.3) | 259.4 (233.5-287.3) | 342.2 (313.2-373.0) | 348.3 (319.2-379.2) | 257.7 (248.2-267.5) |
| *Exposed to insulin (A10A)* | 141.9 (120.3-166.3) | 195.1 (170.6-222.0) | 215.3 (190.4-242.6*)* | 242.3 (216.5-270.3) | 259.8 (233.9-287.7) | 254.3 (228.7-281.9) | 335.3 (306.6-365.9) | 341.4 (312.6-372.1) | 254.5 (245.0-264.2) |
| *Exposed to a* blood *glucose lowering drug (A10B)* | *0.0 (0.0-3.5)* | 0.9 (0.02-4.9) | *0.0 (0.0-3.0)* | 3.1 (0.8-7.9*)* | 1.5 (0.2-5.2) | 4.3 (1.6-9.5) | 5.5 (0.2-10.9) | 5.5 (0.2-10.8) | 2.8 (1.9-4.0) |
| *Exposed to both (A10A + A10B)* | *0.0 (0.0-3.5)* | *0.0 (0.0-3.2)* | *0.0 (0.0-3.0)* | *0.0 (0.0-2.9)* | *0.0 (0.0-2.7)* | 0.7 (0.02-4.0) | 1.4 (0.2-5.0) | 1.4 (0.2-4.9) | 0.5 (0.2-1.3) |

*Supplemental Table 8: Proportion of different insulin and blood glucose lowering drugs prescriptions in the gestational diabetes mellitus group during or after T2, overall and per year between 2012 and 2019.*

| **Gestational diabetes mellitus** | | | | | | | | | |
| --- | --- | --- | --- | --- | --- | --- | --- | --- | --- |
|  | 2012 | 2013 | 2014 | 2015 | 2016 | 2017 | 2018 | 2019 | 2012-2019 |
| **Total pregnancies** | **10,639** | **11,484** | **12,306** | **12,917** | **13,780** | **13,803** | **14,525** | **14,644** | **104'098** |
| Total insulin prescriptions **(**N, (%)) | 358.0 (100.0) | 627.0 (100.0) | 690.0 (100.0) | 848.0 (100.0) | 1008.0 (100.0) | 879.0 (100.0) | 1327.0 (100.0) | 1327.0 (100.0) | 7064.0 (100) |
| *Human insulin (A10AB01, A10AC01)* | *188.0 (52.5, 47.2-57.8)* | *188.0 (30.0, 26.4-33.7)* | *187.0 (27.1, 23.8-30.6)* | *230.0 (27.1, 24.2-30.3)* | *213.0 (21.1, 18.6-23.8)* | *241.0 (27.4, 24.5-30.5)* | *349.0 (26.3, 23.9-28.8)* | *277.0 (20.9, 18.7-23.2)* | *1873.0 (26.5, 25.5-27.6)* |
| *Insulin aspart (A10AB05)* | *128.0 (35,7, 30.8-41.0)* | *227.0 (36.2, 32.4-40.1)* | *239.0 (34.6, 31.1-38.3)* | *232.0 (27.3, 24.4-30.5)* | *243.0 (24.1, 21.5-26.9)* | *184.0 (20.9, 18.3-23.8)* | *288.0 (21.7, 19.5-24.0)* | *325.0 (24.5, 22.2-26.89)* | *1866.0 (26.4, 25.4-27.5)* |
| *Insulin detemir (A10AE05)* | *37.0 (10.3, 7.4-14.0)* | *202.0 (32.2, 28.6-36.0)* | *255.0 (37.0, 33.3-40.7)* | *358.0 (42.2, 38.9-45.6)* | *522.0 (51.8, 48.7-54.9)* | *417.0 (47.4, 44.1-50.8)* | *634.0 (47.8, 45.1-50.1)* | *638.0 (48.1, 45.4-50.8)* | *3063.0 (43.4, 42.2-44.5)* |
| *Insulin glargine (A10AE04)* | *5.0 (1.4, 0.5-32.2)* | *3.0 (0.5, 0.1-1.4)* | *4.0 (0.6, 0.2-1.5)* | *22.0 (2.6, 1.6-3.9)* | *21.0 (2.1, 1.3-3.2)* | *29.0 (3.3, 2.2-4.7)* | *47.0 (3.5, 2.6-4.7)* | *73.0 (5.5, 4.3-6.9)* | *204.0 (2.9, 2.5-3.3)* |
| *Insulin lispro (A10AB04, A10AD04)* | *0.0 (0.0, 0.0-1.0)* | *7.0 (1.1, 0.5-2.3)* | *5.0 (0.7, 0.2-1.7)* | *4.0 (0.5, 0.1-1.2)* | *8.0 (0.8, 0.3-1.6)* | *6.0 (0.7, 0.3-1.5)* | *6.0 (0.5, 0.2-1.0)* | *9.0 (0.7, 0.3-1.3)* | *45.0 (0.6, 0.5-0.9)* |
| *Insulin degludec (A10AE06)* | *0.0 (0.0, 0.0-1.0)* | *0.0 (0.0, 0.0-0.6)* | *0.0 (0.0, 0.0-0.5)* | *1.0 (0.1, 0.003-0-7)* | *1.0 (0.1, 0.003-0.6)* | *1.0 (0.1, 0.003-0.6)* | *2.0 (0.2, 0.02-0.5)* | *1.0 (0.1, 0.002-0.4)* | *6.0 (0.1, 0.03-0.2)* |
| *Insulin glulisine (A10AB06)* | *0.0 (0.0, 0.0-1.0)* | *0.0 (0.0, 0.0-0.6)* | *0.0 (0.0, 0.0-0.5* | *1.0 (0.1, 0.003-0-7)* | *0.0 (0.0, 0.0-0.4)* | *1.0 (0.1, 0.003-0.6)* | *1.0 (0.1, 0.002-0.4)* | *4.0 (0.3, 0.08-0.8)* | *7.0 (0.1, 0.04-0.2)* |
| Total blood glucose lowering drugs prescriptions (N, (%)) | *0.0 (0.0, 0.0-1.0)* | 1.0 (100.0) | 0.0 (100.0) | 4.0 (100.0) | 2.0 (100.0) | 13.0 (100.0) | 11.0 (100.0) | 8.0 (100.0) | 39.0 (100.0) |
| *Metformin* | *0.0 (0.0, 0.0-1.0)* | *0.0 (0.0, 0.0-97.5)* | *0.0 (0.0)* | *3.0 (75.0, 19.4-99.4)* | *2.0 (100.0, 15.8-100.0)* | *12.0 (92.3, 64.0-99.8)* | *11.0 (100.0, 71.5-100.0)* | *8.0 (100.0, 63.1-100.0)* | *36.0 (92.3, 79.1-98.4)* |
| *Sulfonylureas (glibenclamide, glimepiride)* | *0.0 (0.0, 0.0-1.0)* | *1.0 (100.0, 25.0-100.0)* | *0.0 (0.0)* | *1.0 (25.0, 0.6-80.6)* | *0.0 (0.0, 0.0-84.2)* | *1.0 (7.7, 0.2-36.0)* | *0.0 (0.0, 0.0-0.3)* | *0.0 (0.0, 36.9)* | *3.0 (7.7, 1.6-20.9)* |

*Supplemental Figure 1: Distribution of the different insulin and blood glucose lowering drugs prescriptions among continuers during the pre-pregnancy period per calendar year between 2012 and 2019.*


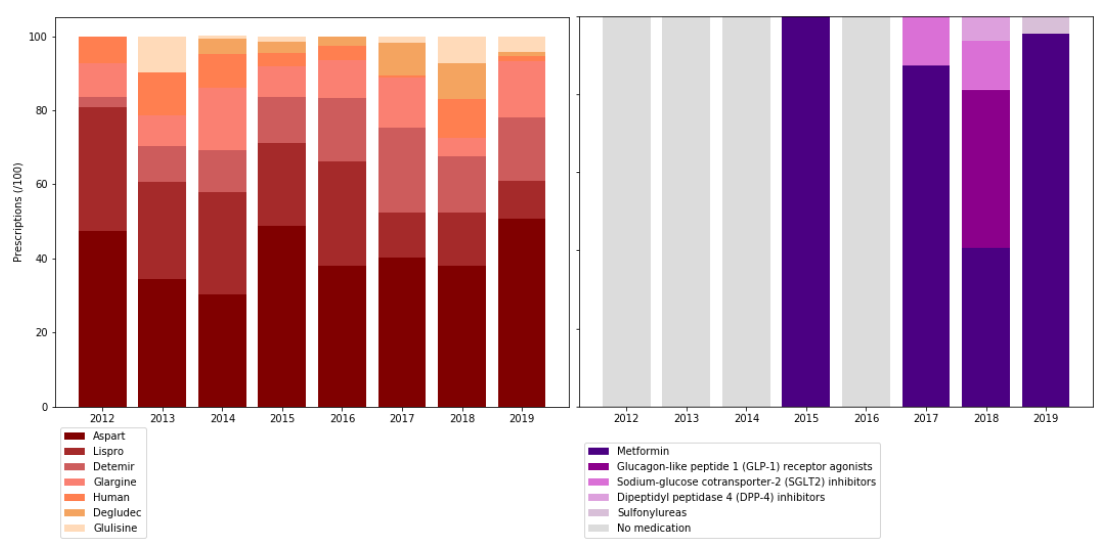


*Supplemental Figure 2: Distribution of the different blood glucose lowering drugs prescriptions among switchers during the pre-pregnancy period per calendar year between 2012 and 2019.*


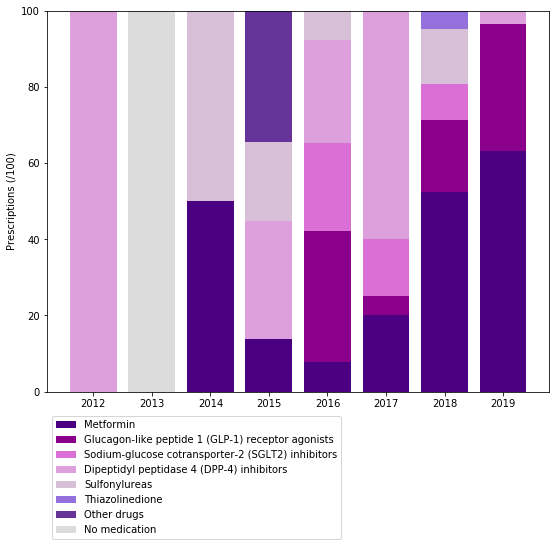
 *Supplemental Figure 3: Distribution of the different insulin and blood glucose lowering drugs prescriptions among discontinuers during the pre-pregnancy period per calendar year between 2012 and 2019.*


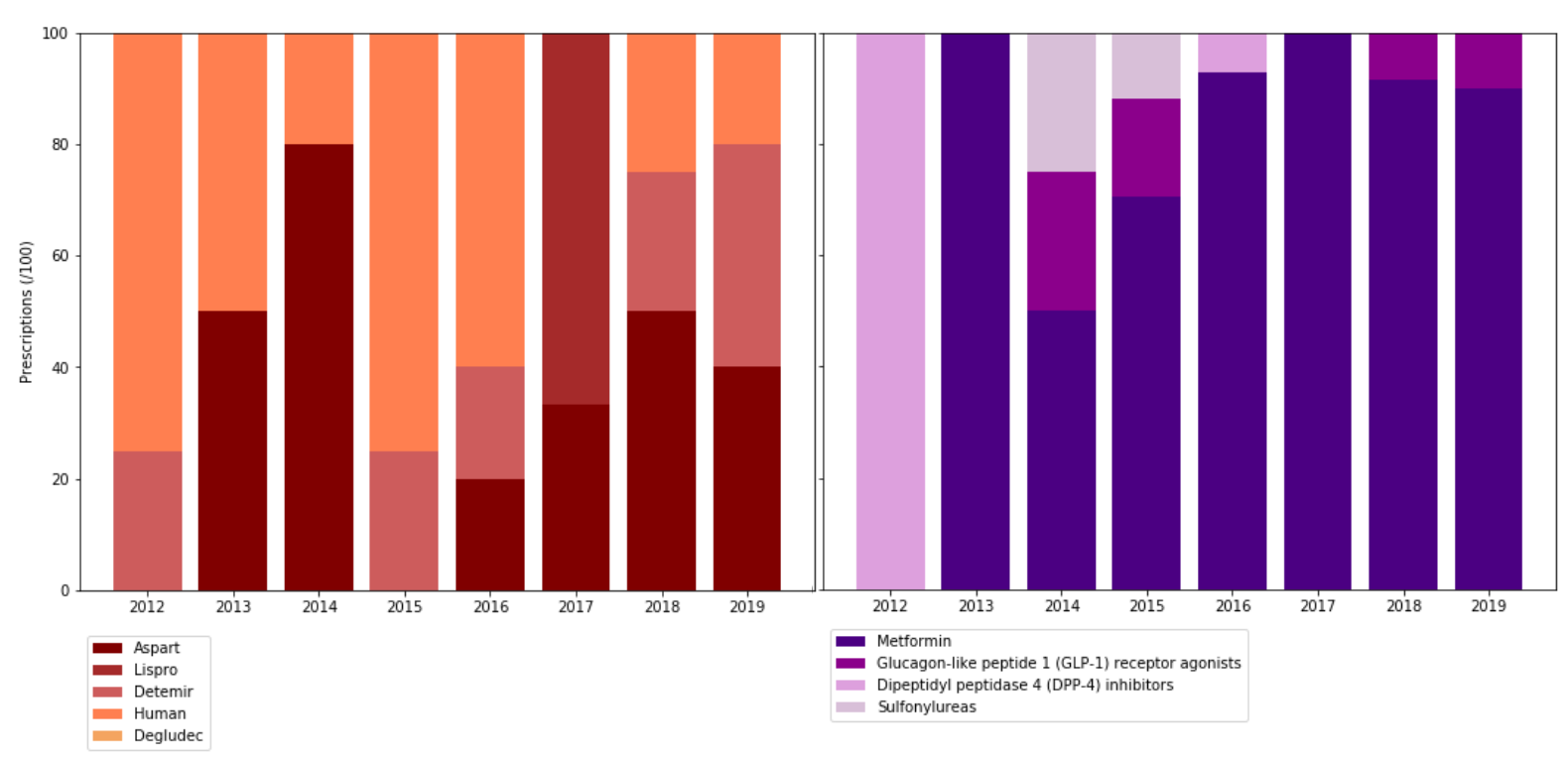


*Supplemental Figure 4: Distribution of the different insulin and blood glucose lowering drugs prescriptions within the gestational diabetes mellitus group during or after T2 per calendar year between 2012 and 2019.*


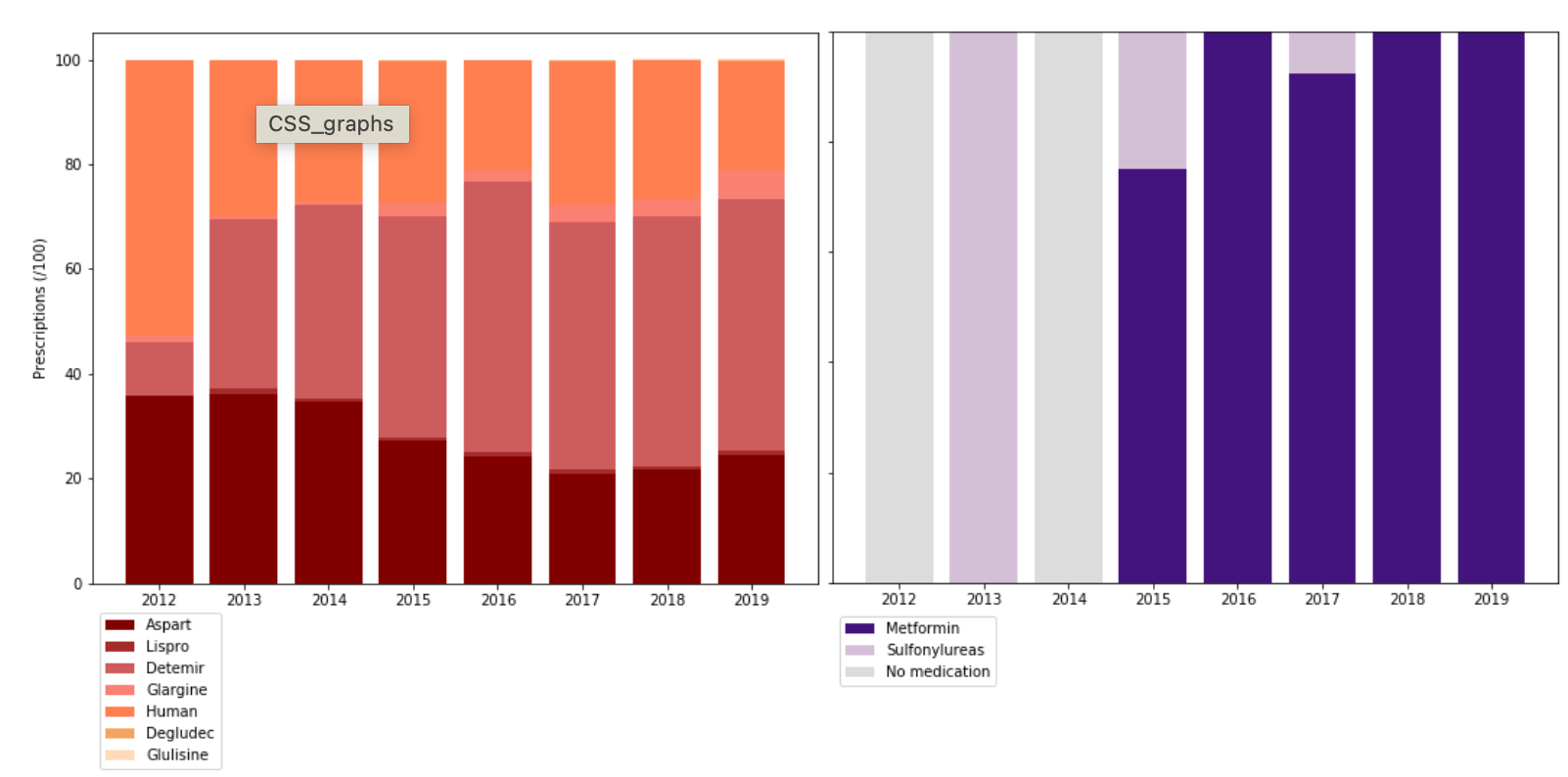

Supplement: Supplementary Materials — Supplemental Table 1: relevant codes used to identify a delivery. Supplemental Table 2: relevant codes to identify a preterm delivery. Supplemental Table 3: prevalence of exposure to insulin, to blood glucose-lowering drugs, or to both insulin and blood glucose-lowering drugs, among continuers, switchers, and discontinuers during the prepregnancy period, overall and per year between 2012 and 2019. Supplemental Table 4: proportion of different insulin and blood glucose-lowering drugs prescriptions within each group of pregestational diabetes mellitus (continuers, switchers) during the prepregnancy period, overall and per year between 2012 and 2019. Supplemental Table 5: ATC codes of medication used in the treatment of infertility. Supplemental Table 6: proportion of different insulin and blood glucose-lowering drugs prescriptions among discontinuers, overall and per year between 2012 and 2019. Supplemental Table 7: prevalence of pregnancies exposed to any ADM, to insulin, to a blood glucose-lowering drugs, or to both insulin and a blood glucose-lowering drug, in the gestational diabetes mellitus group, overall and per year between 2012 and 2019. Supplemental Table 8: proportion of different insulin and blood glucose-lowering drugs prescriptions in the gestational diabetes mellitus group during or after T2, overall and per year between 2012 and 2019. Supplemental Figure 1: distribution of the different insulin and blood glucose-lowering drugs prescriptions among continuers during the prepregnancy period per calendar year between 2012 and 2019. Supplemental Figure 2: distribution of the different blood glucose-lowering drugs prescriptions among switchers during the prepregnancy period per calendar year between 2012 and 2019. Supplemental Figure 3: distribution of the different insulin and blood glucose-lowering drugs prescriptions among discontinuers during the prepregnancy period per calendar year between 2012 and 2019. Supplemental Figure 4: distribution of the diffe [file 4105993.f1.docx]
